# A New Family of Jumonji C Domain-Containing KDM Inhibitors Inspired by Natural Product Purpurogallin

**DOI:** 10.3389/fchem.2020.00312

**Published:** 2020-05-25

**Authors:** José A. Souto, Federica Sarno, Angela Nebbioso, Chiara Papulino, Rosana Álvarez, Jessica Lombino, Ugo Perricone, Alessandro Padova, Lucia Altucci, Ángel R. de Lera

**Affiliations:** ^1^Departamento de Química Orgánica, Facultade de Química and Centro de Investigacións Biomédicas (CINBIO), Universidade de Vigo, Vigo, Spain; ^2^Dipartimento di Medicina di Precisione, Università Degli Studi Della Campania “L. Vanvitelli”, Naples, Italy; ^3^Epi-C srl, Naples, Italy; ^4^Fondazione Ri.MED, Palermo, Italy

**Keywords:** cancer, epigenetics, Jumonji C, histone lysine demethylases, KDM4A enzymes, purpurogallin, benzotropolones, anticancer activity

## Abstract

Aberrant epigenetic modifications are involved in cancer development. Jumonji C domain-containing histone lysine demethylases (KDMs) are found mainly up-regulated in breast, prostate, and colon cancer. Currently, growing interest is focusing on the identification and development of new inhibitors able to block the activity of KDMs and thus reduce tumor progression. KDM4A is known to play a role in several cellular physiological processes, and was recently found overexpressed in a number of pathological states, including cancer. In this work, starting from the structure of purpurogallin **9aa**, previously identified as a natural KDM4A inhibitor, we synthesized two main sets of compound derivatives in order to improve their inhibitory activity against KDM4A *in vitro* and in cells, as well as their antitumor action. Based on the hypothetical biogenesis of the 5-oxo-5*H*-benzo[7]annulene skeleton of the natural product purpurogallin (Salfeld, 1960; Horner et al., 1961; Dürckheimer and Paulus, 1985; Tanaka et al., 2002; Yanase et al., 2005) the pyrogallol and catechol units were first combined with structural modifications at different positions of the aryl ring using enzyme-mediated oxidative conditions, generating a series of benzotropolone analogs. Two of the synthetic analogs of purpurogallin, **9ac** and **9bc**, showed an efficient inhibition (50 and 80%) of KDM4A in enzymatic assays and in cells by increasing levels of its specific targets, H3K9me3/2 and H3K36me3. However, these two compounds/derivatives did not induce cell death. We then synthesized a further set of analogs of these two compounds with greater structural diversification. The most potent of these analogs, **9bf**, displayed the highest KDM4A inhibitory enzymatic activity *in vitro* (IC_50_ of 10.1 and 24.37 μM) in colon cancer cells, and the strongest antitumor action in several solid and hematological human cancer cell lines with no toxic effect in normal cells. Our findings suggest that further development of this compound and its derivatives may lead to the identification of new therapeutic antitumor agents acting through inhibition of KDM4A.

## Introduction

Epigenetic modifications, in other words changes occurring in the phenotype without altering the genotype, are known to be responsible for a myriad of biological processes that can result in the development of fatal diseases such as cancer (Esteller, 2008; DeWoskin and Million, 2013). The main posttranslational structural alterations responsible for epigenetic changes are reversible covalent modifications of histones (including methylation and acetylation of lysine residues), DNA methylation, and regulation of gene expression by non-coding RNAs. The epigenetic enzymes involved in all those reversible changes are potential targets for drug development (Arrowsmith et al., 2012).

Jumonji C (Jmj-C) enzymes, a class of ~60 proteins in humans (Loenarz and Schofield, 2011), are non-heme iron [Fe(II)]- and 2-oxoglutarate (2-OG)-dependent oxygenases that catalyze the demethylation of *N*ε-methylated lysine residues on histone tails, in particular the *N*-terminal tail of histone H3. This epigenetic modification was shown to induce dramatic effects on cell proliferation, causing, among other undesired activities, endocrine deregulation, and cancer development (Loenarz and Schofield, 2008; Handy et al., 2011). The histone lysine demethylase (KDM) 4 (Jmj-D2) subfamily of KDMs (Kooistra and Helin, 2012; Black et al., 2013), which are up-regulated in many cancer cell types (Franci et al., 2014), show a preference for demethylation of tri- and di-*N*ε-methylated H3K9 substrates, with KDM4A-C also acting on H3K36 (Berry and Janknecht, 2013).

**Figure 1:**
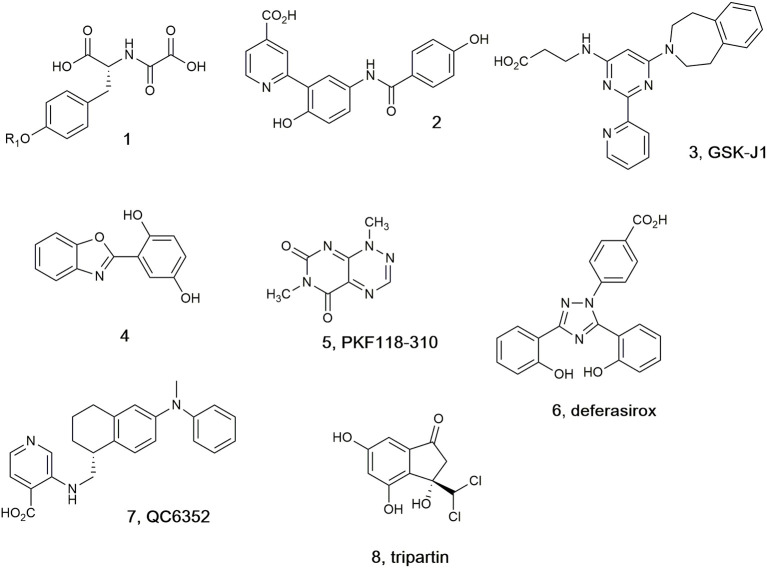
Selected KDM4 inhibitors.

A selection of the few (partially) selective KDM inhibitors identified to date (Lohse et al., 2011a; Rose et al., 2011; Varier and Timmers, 2011; Højfeldt et al., 2013; McAllister et al., 2016), which in general bind in the active site cavity and complex with the Fe(II) cofactor, are illustrated in Figure 1. In addition to the peptide (**1**), which targets the substrate binding site (Lohse et al., 2011b; Woon et al., 2012; Kawamura et al., 2017), other analogs are reported to mimic at least one of the carboxylate groups of the 2-OG co-substrate (Hamada et al., 2009, 2010; Rose et al., 2010). Pyridine carboxylic acid (**2**) was discovered after docking 600,000 fragments into KDM4A, and linking other fragments to the initial 5-aminosalicylate hit (Chen et al., 2017). Compound **2** showed higher potency and selectivity for KDM4C over other KDM subfamilies (Korczynska et al., 2016). Analogs of GSK-J1 (**3**) (Kruidenier et al., 2012) preserving the β-amino acid and changing the 2-(pyridine-2-yl)pyrimidine scaffold were developed and found to exhibit better activity at TNF-α production than parent **3** (Hu et al., 2016). Benzoxazole (**4)** was shown to inhibit KDM6 subfamily member JMJD3 and to induce the cell cycle arrest in the S-phase on A375 melanoma cells (Giordano et al., 2019). KDM4A inhibitor PKF118-310 (**5**), an antagonist of transcription factor 4 (TCF4)/β-catenin signaling, has been recently shown to inhibit KDM4A (Franci et al., 2017). Clinically used iron chelator deferasirox (**6**) was shown to potently inhibit KDM4A *in vitro*, and some of its derivatives with improved cell permeability were shown to significantly upregulate histone trimethylation and act as potent cancer cell growth inhibitors. In addition, deferasirox (**6**) potently inhibited human 2OG-dependent hipoxia inducible factor prolyl hydroxylase activity (Roatsch et al., 2019). Compound QC6352 (**7**) was reported to inhibit KDM4 with high efficacy in breast and colon cancer PDX models (Chen et al., 2017). Tripartin (**8**), the first natural histone lysine demethylase inhibitor (Kim et al., 2013; Guillade et al., 2018), has been recently re-evaluated and shown to cause substantial increase in H3K9me3 levels in HCT-116 cells by Western blot analysis, but either no inhibition or very mild inhibition when tested at 100 μM against isolated KDM4A, B, C, D, or E under standard assay conditions (Guillade et al., 2018).

**Figure 2:**
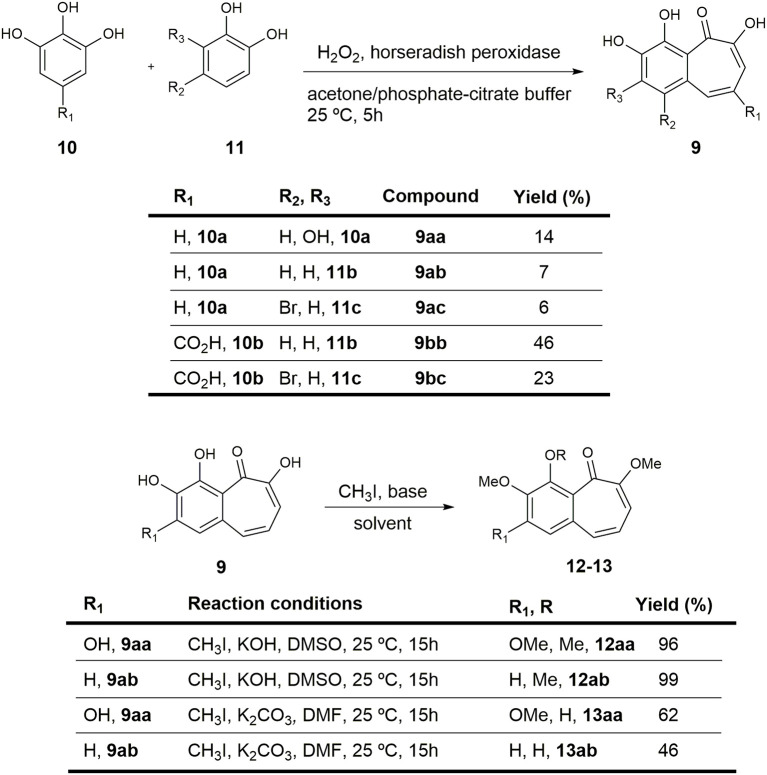
Preparation of purpurogallin **9aa** and sets of analogs.

Using high-throughput screening techniques, we recently screened a library of 10,000 small molecules by *in vitro* KDM4A enzymatic assay (Franci et al., 2017) (see Materials and Methods, and Results) using an automated TECAN robotic station. We identified natural product purpurogallin **9aa** (Figure 2), isolated from nutgalls and oak bark, as an inhibitor of JmjC domain-containing KDMs (Kooistra and Helin, 2012; Berry and Janknecht, 2013; Black et al., 2013). This compound belongs to the family of benzotropolone-containing natural products (Nierenstein and Swanton, 1944; Barltrop and Nicholson, 1948; Takino and Imagawa, 1964; Takino et al., 1964; Arpin et al., 1974; Klostermeyer et al., 2000; Kerschensteiner et al., 2011; Matsuo et al., 2017) and was already known to display antioxidant (Wu et al., 1996) and anticancer activities (Kitada et al., 2003; Leone et al., 2003), and to play a role in the modulation of inflammatory responses (Sang et al., 2004). Purpurogallin and its synthetic analogs were more recently reported to function as inhibitors of Toll-like receptors 1/2 (Cheng et al., 2012), and to modulate mitogen-activated protein kinase 1/2 signaling pathway, reducing esophageal squamous cell carcinoma growth (Xie et al., 2019).

In view of their promising biological activities, we here describe the synthesis of the natural product purpurogallin **9aa** and several of its derivatives, as well as their characterization as KDM inhibitors.

## Materials and Methods

### Chemistry

#### General Remarks

Solvents were dried using a Puresolv® solvent purification system. All other reagents were commercial compounds of the highest purity available. Unless specified, all reactions were carried out under an argon atmosphere and protected from light. Those not involving aqueous reagents were performed in oven-dried glassware. All solvents and anhydrous solutions were transferred through syringes and cannulae previously dried in the oven for at least 12 h and kept in a desiccator. Peroxidase from horseradish Practical Grade I was purchased from Panreac (Castellar del Vallès, Spain, reference number A3791,0025).

Analytical thin-layer chromatography was performed on aluminum plates with Merck Kieselgel 60F_254_ (Merck, Darmstadt, Germany) and visualized by UV irradiation (254 nm) or by staining with an acid solution of phosphomolybdic acid and ethanol. Flash column chromatography was carried out in a Combiflash system using Merck Kieselgel 60 (230–400 mesh) (Merck, Darmstadt, Germany). Infrared (IR) spectra were obtained on a JASCO FTIR 4200 spectrophotometer (JASCO International Co., Tokyo, Japan) from either NaCl window or a diamond ATR probe. Melting points were determined on a Stuart SMP10 apparatus (Stuart Scientific, Stone, UK). High Resolution Mass Spectrometry (HRMS, ESI^+^) was measured with an Apex III FT ICR mass spectrometer (Bruker, Billerica, USA). ^1^H- Nuclear Magnetic Resonance (NMR) spectra were recorded in CDCl_3_, acetone-d_6_, and DMSO-d_6_ at room temperature with a Bruker AMX-400 spectrometer (Bruker, Billerica, USA) operating at 400.16 MHz with residual protic solvent as the internal reference (CDCl_3_, δ = 7.26 ppm, acetone-d_6_, δ = 2.05 ppm, and DMSO-d_6_, δ = 2.50 ppm); chemical shifts (δ) are given in parts per million (ppm) and coupling constants (*J*) are given in Hertz (Hz). The proton spectra are reported as follows: δ (multiplicity, coupling constant *J*, number of protons). ^13^C-NMR spectra were recorded in CDCl_3_, acetone-d_6_, and DMSO-d_6_ at room temperature with the same spectrometer operating at 101.62 MHz with the central peak of CDCl_3_ (δ = 77.00 ppm), acetone-d_6_ (δ = 29.84 ppm), and DMSO-d_6_ (δ = 39.50 ppm) as the internal reference. DEPT-135 pulse sequences were used to aid in the assignment of signals in the ^13^C-NMR spectra.

#### Experimental Procedures and Spectroscopic Data

##### 2,3,4,6-tetrahydroxy-5H-benzo[7]annulen-5-one (purpurogallin 9aa) and 3,4,6-trihydroxy-5H-benzo[7]annulen-5-one (9ab). General procedure for synthesis of the benzotropolone core

Sublimed catechol **11b** (0.11 g, 0.999 mmol) and pyrogallol **10a** (0.188 g, 0.999 mmol) were dissolved in a mixture of acetone and freshly prepared pH 5 phosphate-citrate buffer (1:1 v/v, 28.4 g/L Na_2_HPO_4_/21 g/L citric acid), which contained 0.1 mg of horseradish peroxidase. Freshly prepared 3% H_2_O_2_ (10 mL) was added over 40 min by mechanical syringe and the mixture was stirred at room temperature overnight. The resulting precipitate was filtered off, washed with water (3x), and dried under reduced pressure. The obtained solid was purified by column chromatography to yield 14 mg (7%) of 3,4,6-trihydroxy-5*H*-benzo[7]annulen-5-one **9ab** and 35 mg (14%) of purpurogallin **9aa**.

2,3,4,6-tetrahydroxy-5*H*-benzo[7]annulen-5-one (purpurogallin, **9aa**). Orange solid. ^**1**^**H-NMR** (400 MHz, DMSO-d_6_) δ 7.35 (d, *J* = 11.4 Hz, 1H), 7.07 (d, *J* = 9.4 Hz, 1H), 6.90 (s, 1H), 6.74 (dd, *J* = 11.4, 9.5 Hz, 1H) ppm. ^**13**^**C-NMR** (101 MHz, DMSO-d_6_) δ 182.24 (s), 154.70 (s), 152.31 (s), 151.60 (s), 134.73 (s), 134.33 (d), 133.01 (s), 123.58 (d), 116.52 (d), 114.85 (s), 110.27 (d) ppm. **ATR-FTIR:** ν 3,600–3,400 (br), 3,400–3,200 (br), 2,973 (m), 1,585 (s), 1,419 (s), 1,376 (s), 1,330 (m), 1,196 (s), 1,046 (s) cm^−1^. **HRMS:** (ESI^+^) Calcd. for C_11_H_9_O_5_ ([M+H]^+^), 221.0445; found, 221.0443.

3,4,6-trihydroxy-5*H*-benzo[7]annulen-5-one (**9ab**) Orange solid. ^**1**^**H-NMR** (400 MHz, acetone-d_6_) δ 8.80 (br s, 1H), 8.48 (br s, 1H), 7.59 (d, *J* = 11.4 Hz, 1H), 7.55–7.47 (m, 2H), 7.31 (d, *J* = 9.5 Hz, 1H), 6.89 (dd, *J* = 11.4, 9.5 Hz, 1H) ppm. ^**13**^**C-NMR** (101 MHz, acetone-d_6_) δ 185.04 (s), 155.66 (s), 151.11 (s), 147.11 (s), 136.84 (d), 133.26 (s), 126.31 (d), 124.00 (d), 122.78 (d), 120.94 (s), 119.04 (d) ppm. **ATR-FTIR:** ν 3,600–3,400 (br), 3,400–3,200 (br), 2,918 (m), 1,584 (s), 1,414 (s), 1,327 (s), 1,236 (m), 1,159 (s), 1,093 (s) cm^−1^. **HRMS**: (ESI^+^) Calcd. for C_11_H_9_O_4_ ([M+H]^+^), 205.0495; found, 205.0504.

##### 1-bromo-3,4,6-trihydroxy-5H-benzo[7]annulen-5-one (9ac)

Following the general procedure previously described for the formation of benzotropolones, the reaction of 4-bromocatechol **11c** (0.50 g, 2.66 mmol), pyrogallol **10a** (0.34 g, 2.66 mmol), 3% H_2_O_2_ (21.3 mL), and 0.5 mg of horseradish peroxidase in a mixture of acetone and pH 5 phosphate-citrate buffer (1:1 v/v, 28.4 g/L Na_2_HPO_4_/21 g/L citric acid) afforded 44 mg (6%) of an orange solid identified as 1-bromo-3,4,6-trihydroxy-5H-benzo[7]annulen-5-one (**9ac**). ^**1**^**H-NMR** (400 MHz, acetone-d_6_) δ 9.00 (br s, 1H), 8.07 (d, *J* = 12.0 Hz, 1H), 7.92 (s, 1H), 7.34 (d, *J* = 9.5 Hz, 1H), 7.05 (dd, *J* = 12.0, 9.5 Hz, 1H) ppm. ^**13**^**C-NMR** (101 MHz, acetone-d_6_) δ 185.21 (s), 156.40 (s), 152.09 (s), 147.24 (s), 133.78 (d), 129.80 (s), 127.48 (d), 125.63 (d), 121.80 (s), 118.88 (d), 116.79 (s) ppm. **ATR-FTIR:** ν 3,600–3,400 (br), 3,400–3,200 (br), 1,585 (s), 1,480 (m), 1,419 (s), 1,376 (s), 1,196 (s) cm^−1^. **HRMS:** (ESI^+^) Calcd. for ${\text{C}}_{11}{{\text{H}}_{8}}^{79}$BrO_4_ ([M+H]^+^), 282.9601; found, 282.9608.

##### 3,4,6-trihydroxy-5-oxo-5H-benzo[7]annulene-8-carboxylic acid (9bb)

Following the general procedure previously described for the formation of benzotropolones, the reaction of catechol **11b** (0.29 g, 2.66 mmol), gallic acid **10b** (0.50 g, 1 mmol), 3% H_2_O_2_ (21.3 mL), and 0.5 mg of horseradish peroxidase in a mixture of acetone and pH 5 phosphate-citrate buffer (1:1 v/v, 28.4 g/L Na_2_HPO_4_/21 g/L citric acid) afforded 300 mg (46%) of an orange solid identified as 3,4,6-trihydroxy-5-oxo-5*H*-benzo[7]annulene-8-carboxylic acid (**9bb**). ^**1**^**H-NMR** (400 MHz, DMSO-d_6_) δ 13.55 (br s, 1H), 10.38 (br s, 1H), 9.69 (br s, 1H), 8.33 (d, *J* = 1.5 Hz, 1H), 7.66 (d, *J* = 1.5 Hz, 1H), 7.64 (d, *J* = 8.4 Hz, 1H), 7.49 (d, *J* = 8.4 Hz, 1H) ppm. ^**13**^**C-NMR** (101 MHz, DMSO-d_6_) δ 184.96 (s), 167.64 (s), 153.13 (s), 151.24 (s), 148.64 (s), 138.80 (d), 128.74 (s), 128.25 (d), 123.44 (s), 122.00 (d), 120.36 (s), 115.96 (d) ppm. **ATR-FTIR:** ν 3,600–3,400 (br), 3,400–3,200 (br), 2,973 (m), 1,585 (s), 1,480 (m), 1,419 (s), 1,197 (s) cm^−1^. **HRMS:** (ESI^+^) Calcd. for C_12_H_9_O_6_ ([M+H]^+^), 249.0394; found, 249.0388.

##### 1-bromo-3,4,6-trihydroxy-5-oxo-5H-benzo[7]annulene-8-carboxylic acid (9bc)

Following the general procedure previously described for the formation of benzotropolones, the reaction of 4-bromocatechol **11c** (0.50 g, 2.66 mmol), gallic acid **10b** (0.50 g, 2.66 mmol), 3% H_2_O_2_ (21.3 mL), and 0.5 mg of horseradish peroxidase in a mixture of acetone and pH 5 phosphate-citrate buffer (1:1 v/v, 28.4 g/L Na_2_HPO_4_/21 g/L citric acid) afforded 200 mg (23%) of an orange solid identified as 1-bromo-3,4,6-trihydroxy-5-oxo-5*H*-benzo[7]annulene-8-carboxylic acid (**9bc**). ^**1**^**H-NMR** (400 MHz, DMSO-d_6_) δ 13.46 (br s, 1H), 10.78 (br s, 1H), 9.98 (br s, 1H), 8.87 (d, *J* = 1.4 Hz, 1H), 7.83 (s, 1H), 7.61 (d, *J* = 1.4 Hz, 1H) ppm. ^**13**^**C-NMR** (101 MHz, DMSO-d_6_) δ 185.20 (s), 167.56 (s), 154.06 (s), 151.58 (s), 148.66 (s), 135.46 (d), 126.32 (d), 125.54 (s), 124.84 (s), 121.63 (s), 118.37 (s), 115.26 (d) ppm. **FTIR** (NaCl): ν 3,600–3,200 (br, OH), 2,923 (s), 2,853 (s), 1,699 (s), 1,540 (m), 1,420 (m), 1,310 (m), 1,234 (s) cm^−1^**. HRMS:** (ESI^+^) Calcd. for ${\text{C}}_{12}{{\text{H}}_{8}}^{79}$BrO_6_ ([M+H]^+^), 326.9499; found, 326.9502.

##### 2,3,4,6-tetramethoxy-5H-benzo[7]annulen-5-one (12aa). General procedure for the KOH-mediated methylation of phenols

To a cooled (0°C) suspension of purpurogallin **9aa** (0.015 g, 0.068 mmol) and KOH (0.046 g, 0.818 mmol) in distilled DMSO (2.73 mL) was added CH_3_I (0.034 mL, 0.545 mmol), and the mixture was stirred overnight at room temperature. After this time, the reaction was quenched by addition of water and the mixture was stirred for 20 min. After addition of EtOAc, the organic layer was washed with water (3x) and the combined aqueous layers were extracted with EtOAc (3x). The organic extracts were dried (Na_2_SO_4_) and solvent was removed under reduced pressure to provide 18 mg (96%) of an orange solid identified as 2,3,4,6-tetramethoxy-5*H*-benzo[7]annulen-5-one (**12aa**). **m.p.:** 154–156°C (hex/EtOAc). ^**1**^**H-NMR** (400 MHz, acetone-d_6_) δ 7.07–6.93 (m, 2H), 6.54 (dd, *J* = 10.6, 8.4 Hz, 1H), 6.25 (d, *J* = 8.4 Hz, 1H), 3.97 (s, 3H), 3.95 (s, 3H), 3.86 (s, 3H), 3.78 (s, 3H) ppm. ^**13**^**C-NMR** (101 MHz, acetone-d_6_) δ 185.19 (s), 159.57 (s), 156.13 (s), 153.08 (s), 144.42 (s), 133.25 (s), 129.14 (d), 126.54 (s), 124.24 (d), 107.92 (d), 105.18 (d), 62.78 (q), 60.98 (q), 56.34 (q), 56.21 (q) ppm. **FTIR** (NaCl): ν 3,008 (m), 2,930 (s), 2,848 (s), 1,648 (m), 1,630 (m), 1,580 (s), 1,487 (m), 1,460 (m), 1,394 (m), 1,353 (m), 1,216 (s) cm^−1^. **HRMS:** (ESI^+^) Calcd. for C_15_H_16_NaO_5_ ([M+Na]^+^) 299.0890; found, 299.0895.

##### 3,4,6-trimethoxy-5H-benzo[7]annulen-5-one (12ab)

Following the general procedure previously described for KOH-mediated methylation of phenols, the reaction of **9ab** (0.01 g, 0.05 mmol), KOH (0.033 g, 0.59 mmol), and CH_3_I (0.056 mL, 0.39 mmol) in DMSO (2.0 mL) afforded 12 mg (99%) of an orange solid identified as 3,4,6-trimethoxy-5*H*-benzo[7]annulen-5-one (**12ab**). **m.p.:** 150–152°C (hex/EtOAc). ^**1**^**H-NMR** (400 MHz, acetone-d_6_) δ 7.38 (s, 2H), 6.96 (d, *J* = 11.6 Hz, 1H), 6.43 (dd, *J* = 11.6, 8.5 Hz, 1H), 6.24 (d, *J* = 8.5 Hz, 1H), 3.95 (s, 3H), 3.89 (s, 3H), 3.75 (s, 3H) ppm. ^**13**^**C-NMR** (101 MHz, acetone-d_6_) δ 186.61 (s), 158.36 (s), 153.98 (s), 147.29 (s), 132.64 (s), 129.98 (s), 128.95 (d), 126.37 (d), 122.34 (d), 116.65 (d), 105.76 (d), 62.15 (q), 56.57 (q), 56.25 (q) ppm. **FTIR** (NaCl): ν 2,923 (s), 2,854 (s), 1,730 (m), 1,460 (m), 1,401 (s), 1,284 (m), 1,259 (m), 1,218 (s), 1,027 (s) cm^−1^. **HRMS:** (ESI^+^) Calcd. for C_14_H_14_NaO_4_ ([M+Na]^+^), 269.0784; found, 269.0789.

##### 4-hydroxy-2,3,6-trimethoxy-5H-benzo[7]annulen-5-one (13aa). General procedure for the K_2_CO_3−_mediated methylation of phenols

To a cooled (0°C) suspension of purpurogallin **9aa** (0.01 g, 0.045 mmol) and K_2_CO_3_ (0.075 g, 0.545 mmol) in anhydrous DMF (1.82 mL) was added CH_3_I (0.011 mL, 0.182 mmol), and the mixture was stirred overnight at room temperature. After this time, the reaction was quenched by addition of water and the mixture was stirred for 20 min. The aqueous phase was extracted with EtOAc (3x) and the combined organic layers were washed with water (3x). The organic extracts were dried (Na_2_SO_4_) and the solvent was removed under reduced pressure. The residue was purified by column chromatography to yield 7 mg (62%) of an orange solid identified as 4-hydroxy-2,3,6-trimethoxy-5*H*-benzo[7]annulen-5-one (**13aa**) ^**1**^**H-NMR** (400 MHz, DMSO-d_6_) δ 7.41 (d, *J* = 11.4 Hz, 1H), 7.09 (s, 1H), 6.92 (d, *J* = 9.5 Hz, 1H), 6.83 (dd, *J* = 11.4, 9.5 Hz, 1H), 3.96 (s, 3H), 3.83 (s, 3H), 3.78 (s, 3H) ppm. ^**13**^**C-NMR** (101 MHz, DMSO-d_6_) δ 185.18 (s), 158.14 (s), 157.50 (s), 156.59 (s), 136.21 (s), 135.87 (s), 133.97 (d), 124.15 (d), 117.05 (s), 113.56 (d), 106.20 (d), 59.67 (q), 56.24 (q), 56.02 (q) ppm. **FTIR** (NaCl): ν 3,600–3,200 (br), 3,008 (m), 2,924 (s), 2,851 (s), 1,581 (s), 1,460 (m), 1,411 (s), 1,354 (s), 1,253 (m), 1,214 (s) cm^−1^. **HRMS:** (ESI^+^) Calcd. for C_14_H_15_O_4_ ([M+H]^+^), 263.0914; found, 263.0910.

##### 4-hydroxy-3,6-dimethoxy-5H-benzo[7]annulen-5-one (13ab)

Following the general procedure previously described for K_2_CO_3_-mediated methylation of phenols, the reaction of **9ab** (0.01 g, 0.05 mmol), K_2_CO_3_ (0.081 g, 0.59 mmol), and CH_3_I (0.021 mL, 0.15 mmol) in DMF (2.0 mL) afforded 5 mg (46%) of an orange solid identified as 4-hydroxy-3,6-dimethoxy-5*H*-benzo[7]annulen-5-one (**13ab**). ^**1**^**H-NMR** (400 MHz, DMSO-d_6_) δ 7.61 (d, *J* = 8.7 Hz, 1H), 7.41 (d, *J* = 11.3 Hz, 1H), 7.40 (d, *J* = 8.7 Hz), 6.95 (d, *J* = 9.5 Hz, 1H), 6.69 (dd, *J* = 11.3, 9.5 Hz, 1H), 3.91 (s, 3H), 3.82 (s, 3H) ppm. ^**13**^**C-NMR** (101 MHz, DMSO-d_6_) δ 186.93 (s), 156.37 (s), 153.05 (s), 147.78 (s), 134.80 (d), 130.92 (s), 123.84 (d), 121.55 (d), 120.57 (s), 117.86 (d), 115.02 (d), 56.27 (q), 56.04 (q) ppm. **FTIR** (NaCl): ν 3,600–3,200 (br), 2,925 (s), 2,850 (s), 1,648 (m), 1,630 (m), 1,584 (s), 1,433 (s), 1,210 (m), 1,190 (m), 1,043 (m) cm^−1^. **HRMS:** (ESI^+^) Calcd. for C_13_H_13_O_4_ ([M+H]^+^), 233.0808; found, 233.0805.

##### Methyl 3,4,5-trihydroxybenzoate (10c). General procedure for the esterification of carboxylic acids

To a solution of gallic acid 10b. (5.0 g, 26.6 mmol) in methanol (50.0 mL) was added sulfuric acid, and the solution was stirred at 80°C for 20 h. Then, water was added and the aqueous phase was extracted with EtOAc (3x). The combined organic layers were washed with water (3x), brine (1x), and dried (Na_2_SO_4_), and solvent was evaporated under reduced pressure to obtain 4.3 g (89%) of a white solid identified as methyl 3,4,5-trihydroxybenzoate (**10c**). ^**1**^**H-NMR** (400 MHz, DMSO-d_6_) δ 9.15 (br s, 3H), 6.93 (s, 2H), 3.74 (s, 3H) ppm. ^**13**^**C-NMR** (101 MHz, DMSO-d_6_) δ 166.46 (s), 145.70 (s, 2x), 138.53 (s), 119.46 (s), 108.66 (d, 2x), 51.72 (q) ppm. **ATR-FTIR:** ν 3,600–3,200 (br), 1,669 (s), 1,609 (s), 1,537 (m), 1,461 (m), 1,436 (m), 1,300 (s), 1,254 (s), 1,190 (m), 1,095 (w), 1,033 (s), 1,000 (s) cm^−1^. **HRMS** (ESI^+^): Calcd. for C_8_H_8_O_5_Na ([M+Na]^+^), 207.0261; found, 207.0264.

##### Methyl 1-bromo-3,4,6-trihydroxy-5-oxo-5H-benzo[7]annulene-8-carboxylate (9cc)

Following the general procedure previously described for the formation of benzotropolones, the reaction of 4-bromocatechol **11c** (0.50 g, 2.66 mmol), methyl gallate **10c** (0.50 g, 2.66 mmol), 3% H_2_O_2_ (21.3 mL), and 0.5 mg of horseradish peroxidase in a mixture of acetone and pH 5 phosphate-citrate buffer (1:1 v/v, 28.4 g/L Na_2_HPO_4_/21 g/L citric acid) afforded 612 mg (68%) of an orange solid identified as methyl 1-bromo-3,4,6-trihydroxy-5-oxo-5*H*-benzo[7]annulene-8-carboxylate (**9cc**). ^**1**^**H-NMR** (400 MHz, DMSO-d_6_) δ 8.80 (s, 1H), 7.83 (s, 1H), 7.53 (s, 1H), 3.89 (s, 3H) ppm. ^**13**^**C-NMR** (101 MHz, DMSO-d_6_) δ 184.74 (s), 166.21 (s), 153.83 (s), 151.65 (s), 148.83 (s), 135.41 (d), 126.06 (d), 124.99 (s), 123.40 (s), 121.09 (s), 118.64 (s), 114.44 (d), 53.12 (q) ppm. **ATR-FTIR:** ν 3,600–3,200 (br), 1,719 (s), 1,669 (s), 1,607 (m), 1,434 (m), 1,327 (s), 1,257 (s), 993 (s) cm^−1^. **HRMS** (ESI^+^): Calcd. for ${\text{C}}_{13}{{\text{H}}_{10}}^{79}$BrO_6_ ([M+H]^+^), 340.9655; found, 340.9651.

##### Methyl 1-chloro-3,4,6-trihydroxy-5-oxo-5H-benzo[7]annulene-8-carboxylate (9cd)

Following the general procedure previously described for the formation of benzotropolones, the reaction of 4-chlorocatechol **11d** (0.08 g, 0.54 mmol), methyl gallate **10c** (0.10 g, 0.54 mmol), and 0.5 mg of horseradish peroxidase in a mixture of acetone and pH 5 phosphate-citrate buffer (2.5 mL, 1:1 v/v, 28.4 g/L Na_2_HPO_4_/21 g/L citric acid) with 3% H_2_O_2_ (2.5 mL) afforded 116 mg (72%) of an orange solid identified as methyl 1-chloro-3,4,6-trihydroxy-5-oxo-5*H*-benzo[7]annulene-8-carboxylate (**9cd**). ^**1**^**H-NMR** (400 MHz, DMSO-d_6_) δ 10.85 (s, 1H), 10.05 (s, 1H), 8.80 (s, 1H), 7.65 (s, 1H), 7.54 (s, 1H), 3.88 (s, 3H) ppm. ^**13**^**C-NMR** (101 MHz, DMSO-d_6_) δ 184.54 (s), 166.12 (s), 153.61 (s), 151.30 (s), 148.87 (s), 131.73 (d), 127.24 (s), 123.68 (d), 123.16 (s), 122.52 (s), 120.67 (s), 114.49 (s), 53.07 (q) ppm. **ATR-FTIR:** ν 3,600–3,200 (br), 1,722 (m), 1,592 (w), 1,431 (m), 1,394 (w), 1,235 (s), 995 (s) cm^−1^. **HRMS** (ESI^+^): Calcd. for ${\text{C}}_{13}{{\text{H}}_{10}}^{35}$ClO_6_ ([M+H]^+^), 297.0160; found, 297.0158.

##### Methyl 3,4,6-trihydroxy-1-methyl-5-oxo-5H-benzo[7]annulene-8-carboxylate (9ce)

Following the general procedure previously described for the formation of benzotropolones, the reaction of 4-methylcatechol **11e** (0.08 g, 0.54 mmol), methyl gallate **10c** (0.10 g, 0.54 mmol), and 0.5 mg of horseradish peroxidase in a mixture of acetone and pH 5 phosphate-citrate buffer (2.5 mL, 1:1 v/v, 28.4 g/L Na_2_HPO_4_/21 g/L citric acid) with 3% H_2_O_2_ (2.5 mL) afforded 97 mg (65%) of an orange solid identified as methyl 3,4,6-trihydroxy-1-methyl-5-oxo-5*H*-benzo[7]annul-ene-8-carboxylate (**9ce**). ^**1**^**H-NMR** (400 MHz, DMSO-d_6_) δ 10.29 (s, 1H), 9.76 (s, 1H), 8.53 (d, *J* = 1.5 Hz, 1H), 7.58 (d, *J* = 1.5 Hz, 1H), 7.45 (s, 1H), 3.88 (s, 3H), 2.85 (s, 3H) ppm. ^**13**^**C-NMR** (101 MHz, DMSO-d_6_) δ 185.28 (s), 166.86 (s), 153.46 (s), 149.65 (s), 148.45 (s), 132.99 (d), 132.38 (s), 126.09 (s), 124.60 (s), 121.84 (s), 121.41 (s), 114.74 (d), 53.03 (q), 21.58 (q) ppm. **ATR-FTIR:** ν 3,600–3,200 (br), 1,717 (m), 1,607 (w), 1,434 (m), 1,257 (s), 1,185 (s), 1,119 (s), 993 (s) cm^−1^. **HRMS** (ESI^+^): Calcd. for C_14_H_13_O_6_ ([M+H]^+^), 277.0707; found, 277.0708.

##### Methyl 1-bromo-3,4,6-trimethoxy-5-oxo-5H-benzo[7]annulene-8-carboxylate (13cc). General procedure for the methylation of phenols using dimethyl sulfate

A solution of benzotropolone **9cc** (0.27 g, 0.79 mmol) and K_2_CO_3_ (1.3 g, 9.42 mmol) in DMF (10.7 mL) was added dropwise with dimethyl sulfate (0.79 g, 6.29 mmol), and the reaction mixture stirred at 50°C for 15 h. After this time, the reaction was quenched by addition of water and EtOAc, and the layers were separated. The organic phase was washed with water (3x), the combined aqueous layers were extracted with EtOAc (3x), and the whole organic phase was washed with brine, dried, and the solvent was evaporated under reduced pressure to afford 284 mg (94%) of an orange oil identified as methyl 1-bromo-3,4,6-trimethoxy-5-oxo-5*H*-benzo[7]annulene-8-carboxylate (**13cc**). **m.p.:** 173–175°C (hex/EtOAc). ^**1**^**H-NMR** (400 MHz, CDCl_3_) δ 8.41 (s, 1H), 7.47 (s, 1H), 6.68 (s, 1H), 3.95 (s, 6H), 3.92 (s, 3H), 3.84 (s, 3H) ppm. ^**13**^**C-NMR** (101 MHz, CDCl_3_) δ 186.03 (s), 167.65 (s), 156.79 (s), 154.55 (s), 146.03 (s), 134.22 (s), 132.41 (d), 125.51 (s), 125.09 (s), 120.73 (s), 120.23 (d), 102.27 (d), 62.82 (q), 56.64 (q), 56.38 (q), 53.04 (q) ppm. **FTIR** (NaCl): ν 3,077 (w), 2,991 (w), 2,941 (m), 2,845 (w), 1,715 (s), 1,681 (s), 1,623 (m), 1,577 (s), 1,539 (s), 1,472 (s), 1,445 (s), 1,290 (s), 1,252 (s), 1,223 (s), 1,093 (m), 1,033 (m) cm^−1^. **HRMS** (ESI^+^): Calcd. for ${\text{C}}_{16}{{\text{H}}_{16}}^{79}$BrO_6_ ([M+H]^+^), 383.0125; found 383.0121.

##### Methyl 3,4,6-trimethoxy-5-oxo-1-phenyl-5H-benzo[7]annulene-8-carboxylate (15cf). General procedure for the Suzuki coupling reaction

To a degassed mixture of DMF (1.3 mL) and H_2_O (0.3 mL) was added bromide **13cc** (0.03 g, 0.081 mmol), phenylboronic acid **14f** (0.015 g, 0.122 mmol), Na_2_CO_3_ (0.059 g, 0.56 mmol), and PdCl_2_(dppf) (0.003 g, 0.004 mmol), and the mixture was stirred at 90°C for 90 min. Then, the reaction was quenched by the addition of water. The aqueous layer was extracted with EtOAc (3x). The combined organic layers were dried and the solvent was removed under reduced pressure. The crude residue was purified by column chromatography (silicagel, 0–50% hexane/EtOAc gradient in 20 min) to afford 25 mg (81%) of a white solid identified as methyl 3,4,6-trimethoxy-5-oxo-1-phenyl-5*H*-benzo[7]annulene-8-carboxylate (**15cf**). **m.p.:** 154–156°C (hex/EtOAc). ^**1**^**H-NMR** (400 MHz, CDCl_3_) δ 7.97 (s, 1H), 7.52–7.40 (m, 3H), 7.37–7.32 (m, 2H), 7.15 (s, 1H), 6.69 (s, 1H), 4.02 (s, 3H), 3.96 (s, 3H), 3.86 (s, 3H), 3.75 (s, 3H) ppm. ^**13**^**C-NMR** (101 MHz, CDCl_3_) δ 187.10 (s), 167.92 (s), 156.62 (s), 154.13 (s), 145.89 (s), 140.30 (s), 139.93 (s), 134.15 (s), 132.52 (d), 130.18 (d, 2x), 128.46 (d, 2x), 128.08 (d), 124.80 (s), 123.26 (s), 117.43 (d), 102.32 (d), 62.79 (q), 56.40 (q), 56.30 (q), 52.71 (q) ppm. **FTIR** (NaCl): ν 2,924 (s), 2,852 (m), 1,713 (s), 1,621 (w), 1,569 (m), 1,465 (m), 1,325 (w), 1,254 (s), 1,220 (s) cm^−1^. **HRMS** (ESI^+^): Calcd. for C_22_H_21_O_6_ ([M+H]^+^), 381.1333; found 381.1329.

##### Methyl 3,4,6-trimethoxy-5-oxo-1-4'-trifluoromethyl-phenyl-5H-benzo-[7]annulene-8-carboxylate (15cg)

Following the general procedure previously described for the Suzuki coupling, the reaction of bromide **13cc** (0.031 g, 0.081 mmol), 4-trifluorotolueneboronic acid **14g** (0.023 g, 0.122 mmol), Na_2_CO_3_ (0.059 g, 0.56 mmol), and PdCl_2_(dppf) (0.003 g, 0.004 mmol) in DMF (1.3 mL) and H_2_O (0.3 mL) afforded, after purification by column chromatography (silicagel, hexane/EtOAc 0–50% gradient in 20 min), 25 mg (69%) of a white solid identified as methyl 3,4,6-trimethoxy-5-oxo-1-4'-trifluoromethyl-phenyl-5*H*-benzo[7]annulene-8-carboxylate (**15cg**). **m.p.:** 157–159°C (hex/EtOAc). ^**1**^**H-NMR** (400 MHz, CDCl_3_) δ 7.86 (s, 1H), 7.74 (d, *J* = 8.0 Hz, 2H), 7.47 (d, *J* = 8.0 Hz, 2H), 7.11 (s, 1H), 6.68 (s, 1H), 4.02 (s, 3H), 3.96 (s, 3H), 3.86 (s, 3H), 3.76 (s, 3H) ppm. ^**13**^**C-NMR** (101 MHz, CDCl_3_) δ 186.86 (s), 167.71 (s), 156.85 (s), 154.23 (s), 146.46 (s), 143.98 (s), 138.07 (s), 134.30 (s), 131.59 (d), 130.59 (d, 2x), 130.52 (s, ^2^*J*_C−F_ = 8.1 Hz, 125.46 (d, ^3^*J*_C−F_ = 3.7 Hz), 124.65 (s), 124.08 (s, ^1^*J*_C−F_ = 272.3 Hz), 123.93 (s), 117.29 (d), 102.14 (d), 62.83 (q), 56.47 (q), 56.35 (q), 52.84 (q) ppm. **FTIR** (NaCl): ν 2,925 (s), 2,852 (m), 1,716 (s), 1,619 (w), 1,572 (w), 1,492 (m), 1,461 (m), 1,435 (m), 1,326 (s), 1,256 (m), 1,221 (s) cm^−1^. **HRMS** (ESI^+^): Calcd. for C_23_H_20_F_3_O_6_ ([M+H]^+^), 449.1207; found 449.1200.

##### Methyl 3,4,6-trimethoxy-5-oxo-1-4'-fluoro-phenyl-5H-benzo[7]annulene-8-carboxylate (15ch)

Following the general procedure previously described for the Suzuki coupling, the reaction of bromide **13cc** (0.031 g, 0.081 mmol), 4-fluorophenylboronic acid **14 h** (0.017 g, 0.122 mmol), Na_2_CO_3_ (0.059 g, 0.56 mmol), and PdCl_2_(dppf) (0.003 g, 0.004 mmol) in DMF (1.3 mL) and H_2_O (0.3 mL) afforded, after purification by column chromatography (silicagel, hexane/EtOAc 0–50% gradient in 20 min), 19 mg (59%) of a white solid identified as methyl 3,4,6-trimethoxy-5-oxo-1-4'-fluoro-phenyl-5*H*-benzo[7]annulene-8-carboxylate (**15ch**). **m.p.:** 158–160°C (hex/EtOAc). ^**1**^**H-NMR** (400 MHz, CDCl_3_) δ 7.91 (s, 1H), 7.33–7.28 (m, 2H), 7.16 (t, *J* = 8.6 Hz, 2H), 7.11 (s, 1H), 6.67 (s, 1H), 4.01 (s, 3H), 3.96 (s, 3H), 3.86 (s, 3H), 3.77 (s, 3H) ppm. ^**13**^**C-NMR** (101 MHz, CDCl_3_) δ 187.01 (s), 167.84 (s), 162.68 (s, ^1^*J*_C−F_ = 162.7 Hz), 156.71 (s), 154.15 (s), 146.02 (s), 138.72 (s), 136.25 (s, ^4^*J*_C−F_ = 3.4 Hz), 134.20 (s), 132.13 (d), 131.82 (d, ^3^*J*_C−F_ = 8.1 Hz, 2x), 124.86 (s), 123.48 (s), 117.45 (d), 115.50 (d, ^2^*J*_C−F_ = 21.5 Hz, 2x), 102.20 (d), 62.80 (q), 56.43 (q), 56.32 (q), 52.79 (q) ppm. **FTIR** (NaCl): ν 2,925 (s), 2,853 (m), 1,714 (s), 1,572 (w), 1,489 (m), 1,463 (m), 1,434 (m), 1,254 (m), 1,221 (s) cm^−1^. **HRMS** (ESI^+^): Calcd. for C_22_H_20_FO_6_ ([M+H]^+^), 399.1238; found 399.1234.

##### 1-bromo-3,4,6-trimethoxy-5-oxo-5H-benzo[7]annulene-8-benzamide (16dc). General procedure for the amidation reaction

To a solution of ester **13cc** (0.05 g, 0.13 mmol) in methanol (1.3 mL) and H_2_O (0.4 mL) was added LiOH (0.011 g, 0.26 mmol) and the mixture was stirred at room temperature. Then, the reaction was quenched by addition of a saturated aqueous solution of NaHCO_3_ and the aqueous layer was extracted with EtOAc (2x). The aqueous phase was acidified to pH 1 by addition of an aqueous solution of 10% HCl and then extracted with EtOAc (3x). The combined organic layers were washed with brine (1x), dried, and the solvent was evaporated to isolate a yellow solid that was dissolved in CH_2_Cl_2_ (0.65 mL) and HATU (0.049 g, 0.13 mmol), DIPEA (0.045 mL, 0.26 mmol), and aniline (0.018 mL, 0.20 mmol), and the mixture was stirred at room temperature overnight. The solvent was evaporated under reduced pressure and the residue was purified by column chromatography (silica gel, hexane/EtOAc 0–10% gradient in 15 min) to afford 21 mg (36% combined yield) of a yellow solid identified as 1-bromo-3,4,6-trimethoxy-5-oxo-5*H*-benzo[7]annulene-8-benzamide (**16dc**). **m.p.:** 181–183°C (hex/EtOAc). ^**1**^**H-NMR** (400 MHz, CDCl_3_) δ 7.92 (s, 1H), 7.83 (d, *J* = 1.1 Hz, 1H), 7.66–7.64 (m, 2H), 7.46 (s, 1H), 7.42–7.38 (m, 2H), 7.21–7.16 (m, 1H), 6.52 (s, 1H), 3.96 (s, 3H), 3.94 (s, 3H), 3.82 (s, 3H) ppm. ^**13**^**C-NMR** (101 MHz, CDCl_3_) δ 186.33 (s), 167.40 (s), 157.32 (s), 154.14 (s), 145.95 (s), 138.00 (s), 133.67 (s), 131.46 (s), 129.25 (d, 2x), 126.68 (d), 125.41 (s), 124.92 (d), 120.44 (d, 2x), 120.32 (d), 119.96 (s), 102.08 (d), 62.80 (q), 56.66 (q), 56.51 (q) ppm. **ATR-FTIR:** ν 3,600–3,200 (br), 2,929 (s), 2,852 (m), 1,653 (s), 1,577 (s), 1,537 (m), 1,468 (s), 1,442 (m), 1,292 (s), 1,217 (m), 1,034 (m) cm^−1^. **HRMS** (ESI^+^): Calcd. for ${\text{C}}_{21}{{\text{H}}_{19}}^{79}$BrNO_5_ ([M+H]^+^), 444.0441; found 444.0436.

##### 1-bromo-3,4,6-trihydroxy-5-oxo-5H-benzo[7]annulene-8-carboxylic acid (9bc). General procedure for the BBr_3_-mediated hydrolysis of methyl esters and deprotection of ethers

To a cooled solution (0°C) of ester **9cc** (0.051 g, 0.15 mmol) in CH_2_Cl_2_ (1 mL) was added BBr_3_ (4.65 mL, 4.65 mmol), and the mixture was stirred at room temperature overnight. Then, the mixture was carefully quenched by dropwise addition of a saturated aqueous solution of NaHCO_3_ and the resulting biphasic mixture was stirred for 10 min at room temperature. The aqueous layer was washed with EtOAc (2x) prior to bringing to pH = 1 by addition of 10% HCl. Then, the aqueous layer was extracted with EtOAc (3x), the combined organic layers were washed with brine, dried, and the solvent was evaporated under reduced pressure to render 46 mg (99%) of an orange solid identified as 1-bromo-3,4,6-trihydroxy-5-oxo-5*H*-benzo[7]annulene-8-carboxylic acid (**9bc**).

##### 1-chloro-3,4,6-trihydroxy-5-oxo-5H-benzo[7]annulene-8-carboxylic acid (9bd)

Following the general procedure previously described for the BBr_3_-mediated hydrolysis of methyl esters and deprotection of ethers, the reaction of ester **9cd** (0.13 g, 0.042 mmol) and BBr_3_ (1.31 mL, 1.31 mmol) in CH_2_Cl_2_ (0.44 mL) afforded 9 mg (76%) of an orange solid identified as 1-chloro-3,4,6-trihydroxy-5-oxo-5*H*-benzo[7]annulene-8-carboxylic acid (**9bd**). ^**1**^**H-NMR** (400 MHz, DMSO-d_6_) δ 13.48 (br s, 1H), 10.80 (br s, 1H), 9.99 (s, 1H), 8.90 (s, 1H), 7.68 (s, 1H), 7.62 (s, 1H) ppm. ^**13**^**C-NMR** (101 MHz, DMSO-d_6_) δ 185.25 (s), 167.52 (s), 154.01 (s), 151.14 (s), 148.66 (s), 131.78 (d), 126.94 (s), 124.82 (s), 124.33 (s), 122.91 (d), 121.44 (s), 115.28 (d) ppm. **ATR-FTIR:** ν 3,600–3,200 (br), 2,919 (m), 2,850 (m), 1,692 (s), 1,589 (m), 1,435 (s), 1,401 (m), 1,306 (s), 1,241 (s), 1,119 (m), 1,033 (s), 879 (m) cm^−1^. **HRMS** (ESI^+^): Calcd. for ${\text{C}}_{12}{{\text{H}}_{8}}^{35}$ClO_6_ ([M+H]^+^), 283.0004; found, 283.0005.

##### 1-methyl-3,4,6-trihydroxy-5-oxo-5H-benzo[7]annulene-8-carboxylic acid (9be)

Following the general procedure previously described for the BBr_3_-mediated hydrolysis of methyl esters and deprotection of ethers, the reaction of ester **9ce** (0.09 g, 0.034 mmol) and BBr_3_ (1.05 mL, 1.05 mmol) in DCM (0.35 mL) afforded 9 mg (99%) of an orange solid identified as 1-methyl-3,4,6-trihydroxy-5-oxo-5*H*-benzo[7]annulene-8-carboxylic acid (**9be**). ^**1**^**H-NMR** (400 MHz, DMSO-d_6_) δ 10.23 (s, 1H), 9.71 (s, 1H), 8.62 (s, 1H), 7.66 (s, 1H), 7.47 (s, 1H), 2.61 (s, 3H) ppm. ^**13**^**C-NMR** (101 MHz, DMSO-d_6_) δ 185.25 (s), 167.89 (s), 153.34 (s), 149.57 (s), 148.14 (s), 132.93 (d), 132.10 (s), 126.39 (s), 124.62 (d), 122.88 (s), 121.53 (s), 115.44 (d), 21.61 (q) ppm. **ATR-FTIR:** ν 3,600–3,200 (br), 2,919 (m), 2,850 (m), 1,692 (s), 1,588 (w), 1,435 (s), 1,401 (m), 1,307 (s), 1,241 (s), 1,195 (s), 1,033 (m), 119 (m), 1,033 (s), 879 (m) cm^−1^. **HRMS** (ESI^+^): Calcd. for C_13_H_11_O_6_ ([M+H]^+^), 263.0550; found, 263.0552.

##### 3,4,6-trihydroxy-5-oxo-1-phenyl-5H-benzo[7]annulene-8-carboxylic acid (9bf)

Following the general procedure previously described for the BBr_3_-mediated hydrolysis of methyl esters and ethers, the reaction of **9cf** (0.016 g, 0.042 mmol) and BBr_3_ (1.31 mL, 1.31 mmol) in CH_2_Cl_2_ (0.44 mL) afforded 12 mg (88%) of an orange solid identified as 3,4,6-trihydroxy-5-oxo-1-phenyl-5*H*-benzo[7]annulene-8-carboxylic acid (**9bf**). ^**1**^**H-NMR** (400 MHz, DMSO-d_6_) δ 9.74 (br s, 1H), 8.37 (s, 1H), 7.62 (s, 1H), 7.56–7.42 (m, 3H), 7.41–7.29 (m, 3H) ppm. ^**13**^**C-NMR** (101 MHz, DMSO-d_6_) δ 185.48 (s), 167.64 (s), 153.22 (s), 150.99 (s), 147.72 (s), 140.75 (s), 138.10 (s), 138.08 (s), 135.51 (d), 130.16 (d, 2x), 128.48 (d, 2x), 127.72 (d), 126.19 (s), 123.70 (d), 121.27 (s), 115.82 (d) ppm. **ATR-FTIR:** ν 3,600–3,200 (br), 2,919 (m), 1,692 (s), 1,588 (m), 1,435 (s), 1,307 (s), 1,241 (s), 1,195 (s), 1,119 (m), 1,033 (s), 879 (m) cm^−1^. **HRMS** (ESI^+^): Calcd. for C_18_H_13_O_6_ ([M+H]^+^), 325.0707; found 325.0710.

##### 3,4,6-trihydroxy-5-oxo-1-(4'-trifluoromethyl)-phenyl-5H-benzo[7]annulene-8-carboxylic acid (9bg)

Following the general procedure previously described for the BBr_3_-mediated hydrolysis of methyl esters and ethers, the reaction of ester **15cg** (0.02 g, 0.042 mmol) and BBr_3_ (1.31 mL, 1.31 mmol) in CH_2_Cl_2_ (0.44 mL) afforded 11 mg (63%) of an orange solid identified as 3,4,6-trihydroxy-5-oxo-1-(4'-trifluoromethyl)-phenyl-5*H*-benzo[7]annulene-8-carboxylic acid (**9bg**). ^**1**^**H-NMR** (400 MHz, DMSO-d_6_) δ 10.53 (br s, 1H), 9.82 (br s, 1H), 8.29 (s, 1H), 8.05 (d, *J* = 8.0 Hz, 2H), 7.61 (s, 1H), 7.50 (d, *J* = 8.0 Hz, 2H), 7.41 (s, 1H) ppm. ^**13**^**C-NMR** (101 MHz, DMSO-d_6_) δ 185.54 (s), 167.44 (s), 167.02 (s), 153.49 (s), 151.36 (s), 147.82 (s), 145.11 (s), 136.92 (s), 135.09 (d), 130.51 (d, 2x), 129.97 (s), 129.40 (d, 2x), 124.55 (s, ^1^*J*_C−F_ = 282.2 Hz), 123.46 (d), 121.31 (s), 119.49 (s), 115.53 (d) ppm. **ATR-FTIR:** ν 2,926 (m), 2,850 (m), 1,713 (s), 1,681 (m), 1,622 (w), 1,577 (w), 1,468 (m), 1,441 (m), 1,389 (w), 1,287 (s), 1,247 (s), 1,225 (s), 1,035 (w) cm^−1^. **HRMS** (ESI^+^): Calcd. for C_19_H_12_F_3_O_6_ ([M+H]^+^), 393.0581; found 393.0580.

##### 3,4,6-trihydroxy-5-oxo-1-(4'-fluoro)-phenyl-5H-benzo[7]annulene-8-carboxylic acid (9bh)

Following the general procedure previously described for the BBr_3_-mediated hydrolysis of methyl esters and ethers, the reaction of ester **15ch** (0.016 g, 0.040 mmol) and BBr_3_ (1.31 mL, 1.31 mmol) in CH_2_Cl_2_ (0.44 mL) afforded 10 mg (73%) of an orange solid identified as 3,4,6-trihydroxy-5-oxo-1-(4'-fluoro)-phenyl-5*H*-benzo[7]annulene-8-carboxylic acid (**9bh**). ^**1**^**H-NMR** (400 MHz, DMSO-d_6_) δ 10.50 (br s, 1H), 9.77 (br s, 1H), 8.31 (s, 1H), 7.62 (s, 1H), 7.52–7.07 (m, 5H) ppm. ^**13**^**C-NMR** (101 MHz, DMSO-d_6_) δ 185.48 (s), 167.52 (s), 161.69 (s, ^1^*J*_C−F_ = 244.8 Hz), 153.32 (s), 151.08 (s), 147.73 (s), 137.05 (s, ^4^*J*_C−F_ = 3.3 Hz), 136.88 (s), 135.25 (d), 132.21 (d, ^3^*J*_C−F_ = 8.3 Hz, 2x) 126.27 (s), 123.75 (d), 123.13 (s), 121.26 (s), 115.67 (d), 115.36 (d, ^2^*J*_C−F_ = 21.5 Hz, 2x) ppm. **ATR-FTIR:** ν 2,924 (s), 2,851 (m), 1,714 (s), 1,681 (m), 1,622 (w), 1,576 (s), 1,468 (s), 1,442 (s), 1,389 (w), 1,288 (w), 1,249 (s), 1,225 (s), 1,095 (w), 1,035 (m) cm^−1^. **HRMS** (ESI^+^): Calcd. for C_18_H_12_FO_6_ ([M+H]^+^), 343.0613 found 343.0614.

##### 1-bromo-3,4,6-trihydroxy-5-oxo-5H-benzo[7]annulene-8-benzamide (9dc)

Following the general procedure previously described for BBr_3_-mediated demethylation, the reaction of amide **16dc** (0.02 g, 0.043 mmol) and BBr_3_ (1.33 mL, 1.33 mmol) in CH_2_Cl_2_ (0.44 mL) afforded 8 mg (47%) of an orange solid identified as 1-bromo-3,4,6-trihydroxy-5-oxo-5*H*-benzo[7]annulene-8-benzamide (**9dc**). ^**1**^**H-NMR** (400 MHz, DMSO-d_6_) δ 10.66 (br s, 1H), 10.56 (s, 1H), 10.11 (br s, 1H), 8.44 (s, 1H), 7.87 (s, 1H), 7.74 (d, *J* = 8.4 Hz, 2H), 7.47 (s, 1H), 7.37 (t, *J* = 8.0 Hz, 2H), 7.12 (t, *J* = 7.3 Hz, 1H) ppm. ^**13**^**C-NMR** (101 MHz, DMSO-d_6_) δ 185.09 (s), 166.80 (s), 154.49 (s), 151.21 (s), 147.88 (s), 139.05 (s), 132.44 (d), 130.66 (s), 128.66 (d, 2x), 126.50 (d), 125.93 (s), 123.88 (d), 121.46 (s), 120.24 (d, 2x), 117.50 (s), 115.53 (d) ppm. **ATR-FTIR:** ν 3,600–3,200 (br), 2,924 (s), 2,853 (s), 1,713 (s), 1,681 (m), 1,622 (w), 1,577 (w), 1,491 (m), 1,460 (m), 1,443 (w), 1,290 (w), 1,035 (w) cm^−1^. **HRMS** (ESI^+^): Calcd. for ${\text{C}}_{18}{{\text{H}}_{13}}^{79}$BrNO_5_ ([M+H]^+^), 401.9972; found 401.9971.

## Biology

### Cell Lines

Human colon cancer HCT-116 and HT-29, breast cancer MCF7, lung cancer A549, and pancreatic cancer MiaPaCa cells (all from ATCC, VA, USA) were propagated in Dulbecco's Modified Eagle's Medium (Euroclone, Milan, Italy) with 10% fetal bovine serum (FBS) (Euroclone), 2 mM L-glutamine (Euroclone), and antibiotics (100 U/mL penicillin-streptomycin) (Euroclone). Acute promyelocytic leukemia (NB4), histiocytic lymphoma (U937), hepatocellular carcinoma (Hep-G2), and mesenchymal progenitor (Mepr2B) cell lines were propagated in RPMI 1640 medium containing 4.5 g/L glucose (Euroclone) supplemented with 10% FBS (Euroclone), 100 U/mL penicillin-streptomycin (Euroclone), and 2 mM L-glutamine (Euroclone).

### KDM4B/C/D-GST Recombinant Enzyme Purification

KDM4B/C/D-GST enzymes were purified from *Escherichia coli* BL21 bacteria after transfection with pGEX-4T-1 plasmid. One bacteria colony was grown in LB Broth medium (Sigma-Aldrich, Milan, Italy) supplemented with antibiotics (100 μg/mL ampicillin, Sigma) overnight at 37°C. The enzyme expression was induced by isopropyl-β-D-1-thiogalactopyranoside (IPTG, AppliChem, Cinisello Balsamo, Milan, Italy) at 200 μM for 7 h. The bacteria were centrifuged at 3,000 rpm (Beckman Coulter J-25 centrifuge, Milan, Italy) for 20 min and after the pellet was lysated by sonication (Bioruptor, Diagenode, Liège, Belgium) in lysis buffer containing PBS with 1 mM dithiothreitol (DTT, AppliChem) and cOmplete™ Protease Inhibitor Cocktail 1x (Merck, Sigma). The sonication was performed in 10 cycles for 45 s at 14,000 MHz with intervals of 30 s between each sonication. The bacteria suspension was incubated for 15 min in ice in 0.1% of Triton-X100 (Applichem) solution and centrifuged at 13,000 rpm for 30 min and then filtered with a filter of 0.45 μm. The enzymes were purified using a GSTrap 4B columns (GE Healthcare Life Sciences). The columns were equilibrated with 20 mL of lysis buffer. The elution carried out with elution buffer [50 mM Tris-HCl at pH 8.0, 1 mM of DTT, 20 mM L-glutathione reduced (AppliChem)]. The purified recombinant enzymes were dialyzed in water solution (50 mM Tris-HCl pH 8.0, 100 mM NaCl (Sigma), 1 mM DTT and protease inhibitors) overnight at 4°C. The day after, the samples were cryopreserved in 20% glycerol (SigmaAldrich).

### Enzymatic Assay

Assay was performed as previously described using a KDM4A Inhibitor Enzymatic Assay KIT (Epi-C, Naples, Italy) (Franci et al., 2017; Sarno et al., 2018), and by JMJD2A (KDM4A) Homogeneous Assay Kit (#50413; BPS Bioscience, Milan, Italy). In KDM4A Inhibitor Enzymatic Assay KIT by Epi-C, the compounds (6 μL) were incubated for 30 min at 37°C with 24 μL of enzymatic solution in a 96-well black half-area plate. After 30 min at room temperature with the developer, fluorescence was measured with a TECAN M-200 reader (Männedorf, Switzerland) at excitation wavelength of 370 nm and emission wavelength of 470 nm. The same protocol was used to evaluate the activity of **9bf** against KDM4B/C and D. In AlphaLisa assay KDM4A enzyme was incubated with **9bf** at 10 different concentrations, and the assay was done following the protocol of the kit. The IC_50_ was calculated by GraphPad Prism 7.0 software (GraphPad Software, Inc., San Diego, CA, United States). The experiments were performed in triplicate.

### Reagents

All compounds for biological studies were re-suspended in DMSO (Sigma-Aldrich, Milan, Italy) at 50 μM.

### Protein Histone Extraction

MCF7 cells were incubated with compounds **9ac** and **9bc** at 25 μM for 24 h, and with 10 μM **9bf** for 24 h, 28 h and 24 h + 24 h (compounds were added again 24 h post 24 h stimulation). PKF118-310 (**5)** (1 μM) and QC6352 (**7**) (10 μM) were used as positive controls. Cells were then lysed in Triton Extraction Buffer (TEB) containing PBS with 0.5% Triton X-100 (v/v), 2 mM phenylmethylsulfonyl fluoride, and 0.02% (w/v) NaN_3_ at a cell density of 10^7^ cells/mL for 10 min on ice and centrifuged (2,000 rpm at 4°C for 10 min). The supernatant was removed, and the pellet washed with half the volume of TEB and once more centrifuged. The pellet was suspended in 0.2 N HCl overnight at 4°C on a rolling table. The samples were centrifuged at 2,000 rpm for 10 min at 4°C and the supernatant was recovered. The histone protein was determined using a Bradford assay (Bio-Rad, Milan, Italy).

### Western Blot

Western blot analysis was performed following the recommendations of antibody suppliers, loading 8 μg of histone extracts on 15% polyacrylamide gels. Antibodies used were H3K9me3, H3K36me3, H3K4me1, H3K4me2, H3K4me3, and H3K27me2 (Diagenode), and histone H4 (Abcam, Cambridge, UK). Semi-quantitative analysis was performed using ImageJ software.

### Cell Cycle and Cell Death Analysis

HCT-116 cells (1 × 10^5^ cells/well) were plated in a six-well plate and treated with **9ac** and **9bc** at 25 μM for 24 h, and with **9bf** at 25 μM for 12 h and 24 h. In addition, NB4 and U937 cells (2 × 10^5^ cells/mL) were incubated with the same compound at 1, 5, and 10 μM for 12, 24, and 48 h. SAHA (vorinostat, 5 μM) and PKF118-310 (**5**) (1 μM) were used as controls for 24 h. After treatment, cells were collected, then centrifuged (1,200 rpm for 5 min) and suspended in a solution containing 1 × PBS, 0.1% sodium citrate, 0.1% NP40, and 50 mg/mL propidium iodide. After 20 min of incubation at room temperature in the dark, cell cycle was evaluated by FACS (FACSCalibur, BD Biosciences, CA, USA) and analyzed with ModFit v3 software (Verity Software House). Cell death was measured as a percentage of cells in pre-G1 phase and analyzed by FACS with Cell Quest software (BD Biosciences, Milan, Italy).

### MTT Assay

The viability of cells was determined using MTT [3-(4,5-dimethylthiazol-2-yl)-2,5-diphenyltetrazolium bromide] assay. Briefly, HT-29, MCF7, A549, MiaPaCa, Hep-G2, and Mepr2B cells (5 × 10^4^ cells/well) were plated in a 24-well plate and treated, in duplicate, with **9bf** at 10 different concentrations (50–0.09 μM) for 72 h. MTT solution was added for 3 h at 0.5 mg/mL, the purple formazan crystals were dissolved in DMSO (Sigma-Aldrich), and the absorbance was read at a wavelength of 570 nm with a TECAN M-200 reader (Tecan).

### Statistical Analysis

All experiments reported in text represent the mean of three independent experiments with an error bar indicating standard deviation. Differences between the treatments vs. control were analyzed using GraphPad Prism 7.0 software. Statistical comparison was performed by applying one-way analysis of variance (ANOVA) and Dunnett's multiple-comparison test. Differences between groups were considered to be significant at a *p*-value of < 0.05.

### Ligand Preparation

The ligands were prepared by LigPrep, a tool of MAESTRO software, using OPLS3e as force field (Jorgensen and Tirado-Rives, 1988; Jorgensen et al., 1996; Roos et al., 2019) and ionizer to generate all the possible states at pH 7.0 ± 0.2. Desalted and generated tautomers were flagged on, and at most 32 conformers per ligand were generated.

### Ligand Docking

A crystal structure of the protein KDM4A in complex with a ligand (PDB ID: 5VGI, Resolution: 2.7 Å) was chosen from the Protein Data Bank (PDB) (Berman et al., 2000) for the set-up of our model. Protein was prepared via Protein Preparation Wizard Maestro tool (Madhavi Sastry et al., 2013). Epik was used to generate het states at pH 7.4 ± 0.2, deleting waters beyond 5.00 Å and then side chains protonation state was optimized using ProPKa3 at pH 7.4 ± 0.2 (Shelley et al., 2007). The grid was generated with Glide, setting a scaling factor of 1.0, and identifying the grid centroid onto the ligand. Molecular docking was carried out using Glide software released by Schrödinger (release 2018–4) (Friesner et al., 2004, 2006; Halgren et al., 2004). The simulation was performed in extra precision (XP), using OPLS3e as force field (Jorgensen and Tirado-Rives, 1988; Sherman et al., 2006) considering ligands as Flexible and including Epik state penalties to docking score. In this step, the van der Waals radii scaling factor was set as 0.8 and the partial charge cut-off was of 0.15. The searching algorithm on our model was tested using a cognate docking of the co-crystallized ligand, and we obtained an RMSD value of 0.5813. XP docking were performed with constraints. As constraints 2 hydrogen bonds on Tyr132 and Lys206, a positional and a metal constraint were fixed, setting 2 of them as mandatory.

### MM GBSA

The binding affinity was also investigated, starting from XP docking poses with Prime MM-GBSA by Schrödinger software, using VSGB as solvation model and OPLS3e as force field (Li et al., 2011).

### Induced-Fit Docking

Induced-fit docking (Sherman et al., 2006) was carried out. A standard protocol was used, generating up to 20 poses (sample ring conformations with an energy window of 2.5 Kcal/mol). The receptor and ligand van der Waals scaling factors were set to 0.5. Prime was used for the refinement and the re-docking was performed in extra precision.

## Results

### Synthesis of Direct Analogs of Purpurogallin

Most of the previously described methodologies for the preparation of benzotropolone-containing natural products rely on enzyme-mediated oxidative condensations of catechol (**11**) and pyrogallol (**10**) derivatives (Bentley, 2008; Kerschensteiner et al., 2011; Cheng et al., 2012). However, alternative protocols for the synthesis of this family of compounds allow for further generation of structural diversity (Plüg and Friedrichsen, 1992; Fukui et al., 2012; Arican et al., 2017).

In order to obtain straightforward access to an initial set of compounds, we subjected different pyrogallol and catechol derivatives to selected reaction conditions as previously reported (Bentley, 2008; Kerschensteiner et al., 2011; Cheng et al., 2012). We were able to synthesize the natural product and some analogs, albeit in modest yields.

First, to assess whether increased reactivity of the catechol ring (**11**, Figure 2) could improve enzymatic activity against KDM4A, a *para*-bromine group was added (**9ac** and **9bc**). In addition, a carboxylic acid was incorporated on the pyrogallol skeleton (**10**) in order to evaluate whether this group could influence the interaction with the receptor-binding pocket by hydrogen bonding interactions (**9bb** and **9bc;** Figure 2, upper panel). Additionally, the catechol ring was substituted in different ways with a methoxy group to improve the cell membrane permeability against the cellular membrane (Figure 2, lower panel).

### Purpurogallin Analogs Inhibit KDM4A in Enzymatic Assays and in Cells

**Figure 3:**
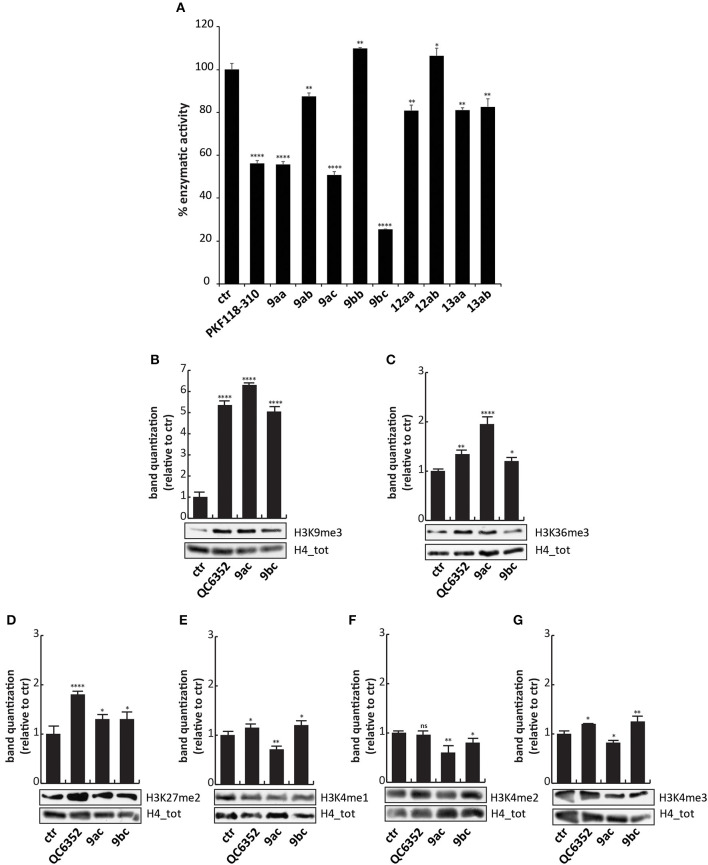
Biological characterization. **(A)** *In vitro* KDM4A enzymatic assay of the compounds at 50 μM. PKF118-310 (**5**) was used as a positive control at 10 μM. **(B–G)** Western blot analysis of H3K9me3 **(B)**, H3K36me3 **(C)**, H3K27me2 **(D)**, H3K4me1 **(E)**, H3K4me2 **(F)**, and H3K4me3 **(G)** in MCF7 breast cancer cells incubated for 24 h with **9ac** and **9bc** at 25 μM. QC6352 (**7**) was used as positive controls for 24 h at 10 μM. H4 antibody was used to normalize total proteins loaded. Bands were quantized using ImageJ software. ****p-value ≤ 0.0001, ***p-value ≤ 0.001, **p-value ≤ 0.01, *p-value ≤ 0.05, ^*ns*^*p*-value > 0.05 vs control cells.

To evaluate the ability of these synthesized compounds to inhibit KDM4A and to modulate methylation levels of lysine 9 and 36, we tested the molecules by *in vitro* enzymatic assay and in tumor cell lines. The effect of the compounds was noticeable at 50 μM on previously purified recombinant KDM4A (Franci et al., 2014). This enzymatic assay reveals the activity of the compounds by the synthesis of a fluorescent molecule (showing excitation wavelength of 370 nm and emission wavelength of 470 nm), directly proportional to enzyme activity (Franci et al., 2017). Compounds **9ac** and **9bc** exhibited a very promising inhibitory activity (stronger than **9aa**), blocking KDM4A and therefore reducing synthesis of the fluorescent compound. Specifically, **9bc** showed a greater enzymatic inhibition than PKF118-310 (**5**), which was used as positive control (Figure 3A and Supplementary Figure 1).

These findings prompted us to hypothesize that the higher activity against KDM4A exerted by compounds **9ac** and **9bc** compared to **9aa** was mainly due to the electron-donating effect of the halogen placed at the *para*-position relative to one of the hydroxyl groups. Furthermore, the presence of the carboxylic acid group in **9bc** improved its activity compared to **9ac**, blocking KDM4A enzymatic activity by 80%. This finding suggests that carboxylic acid might be involved in additional hydrogen-bonding interactions. Transformation of the phenol OH to the aryl methyl ether (OMe) may also have led to the decreased activity of these derivatives.

To characterize their activity, analogs **9ac** and **9bc** were tested at 25 μM for 24 h on MCF7 cells, in which high levels of *KDM4* gene are expressed (Supplementary Figure 3), and their effect was compared to those of KDM4A inhibitor QC6352 (**7**) (Chen et al., 2017) used at 10 μM as positive control. Western blot analysis showed that **9ac** induced a considerable increase in methylation of lysine 9 (H3K9me3) and lysine 36 in histone 3 (H3K36me3), considered *bona fide* KDM4A targets (Figures 3B,C); instead **9bc** only altered the methylation status of lysine 9. The data obtained in cells, differently to enzymatic results, showed that compound **9ac** is a more potent inhibitor than **9bc**, and is able to strongly increase H3K9 trimethylation levels. The decreased activity of compound **9bc** might be due to the presence of the carboxylic acid, which might likely decrease its rate of passive diffusion across the cell membrane.

Since KDM enzymes are known to have a similar structure, it is difficult to obtain compounds displaying greater selectivity toward one isoform. Therefore, the levels of other KDM targets such as H3K4me1/2/3 and H3K27me2 were evaluated after **9ac** and **9bc** treatment (Figures 3D–G). The compounds did not induce variation in the methylation of lysine 4 and 27, showing greater selectivity than compound QC6352 (**7**).

**Figure 4:**
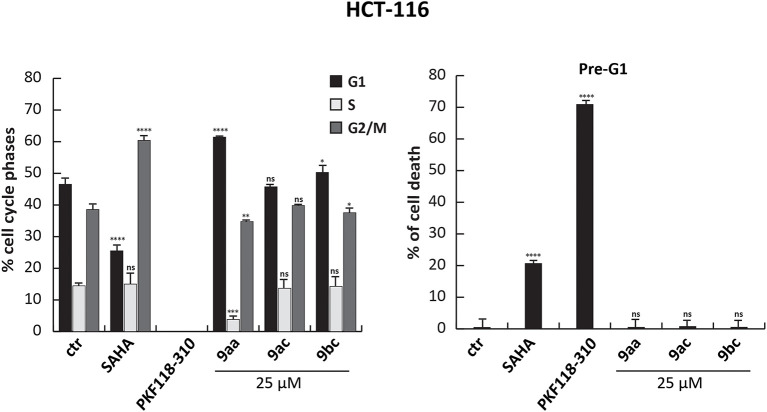
Cell cycle analysis of **9aa**, **9ac**, and **9bc**. HCT-116 cells were treated with **9aa**, **9ac**, and **9bc** for 24 h at 25 μM. SAHA and PKF118-310 (**5**) were used as positive controls for 24 h at 5 and 10 μM, respectively. Left panel shows the percentage of cell cycle phases and right panel shows the percentage of cell death evaluated by propidium iodide. The analysis was performed using ModFit v3 software. ****p-value ≤ 0.0001, ***p-value ≤ 0.001, **p-value ≤ 0.01, *p-value ≤ 0.05, ^*ns*^*p*-value > 0.05 vs control cells.

However, when we tested the ability of these compounds to induce cell cycle modification and cell death, we did not observe any variation. HCT-116 cells treated with **9aa, 9ac**, and **9bc** for 24 h at 25 μM did not show any variation in cell cycle phases compared to induction with SAHA, and PKF18-310 (**5**), which induced 20 and 73% of cell death, respectively (Figure 4). We therefore decided to further modify the benzotropolone groups in order to improve their anticancer activity.

### Synthesis of a Set of 9ac and 9bc Analogs

**Figure 5:**
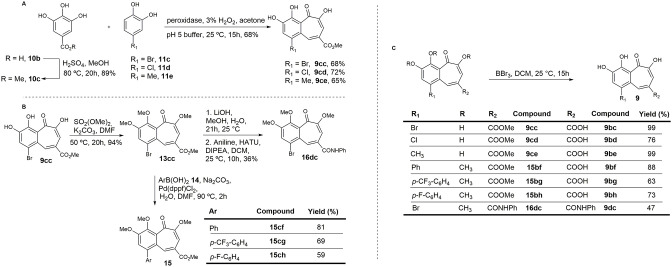
Synthesis of the second set of purpurogallin analogs. **(A)** Synthesis of the benzotropolone core. **(B)** Derivatization of 13cc. **(C)** Hydrolysis of methyl esters and ethers.

Based on preliminary results from enzymatic assays and in-cell experiments, and on the effect induced by the carboxylic acid attached to the pyrogallol ring on permeability, a new synthetic approach was designed in order to obtain benzotropolones with broader structural diversity and higher yields. A second set of compounds was synthesized in which an ester or amide group was introduced at C4 of the benzotropolone scaffold (R_2_) (Figure 5). Furthermore, several substituents were incorporated at C5 (R_1_). For compounds **9cc**, **9cd**, and **16dc**, the presence of a halogen, Br or Cl, was maintained. For compounds **15bf**, **15bg**, and **15bh**, this position was variously replaced with a *para*-substituted phenyl group in order to evaluate whether the presence of further π-π interactions could increase the binding efficacy. Hence, methyl gallate **10c**, prepared in 89% yield by Fischer esterification of commercially available gallic acid **10b**, was condensed with 4-bromocatechol **11c** to provide the corresponding benzotropolone **9cc** in 68% yield. Under the same reaction conditions, chloro- and methyl-substituted analogs (**9cd** and **9ce**) were prepared by condensation of methyl gallate **10c** with 4-chloro- and 4-methylcatechol (**11d** and **11e**) in 72 and 65% yield, respectively (Figure 5A). Compound **9cc** was fully methylated to afford derivative **13cc** in 94% yield, and further derivatized by means of Suzuki cross-coupling reactions with different arylboronic acids to render aryl-substituted compounds **15** in moderate yield (Arican et al., 2017). Orthogonal hydrolysis of the ester functionality and subsequent condensation of the carboxylic acid with aniline was also performed to generate amide **16dc** in 36% yield (Figure 5B). Lastly, all protecting groups were simultaneously removed by reaction with BBr_3_ to afford the target compounds in good yields.

### Compound 9bf Strongly Inhibits KDM4A and Mimics the 2-Oxoglutarate in the Catalytic Site

**Figure 6:**
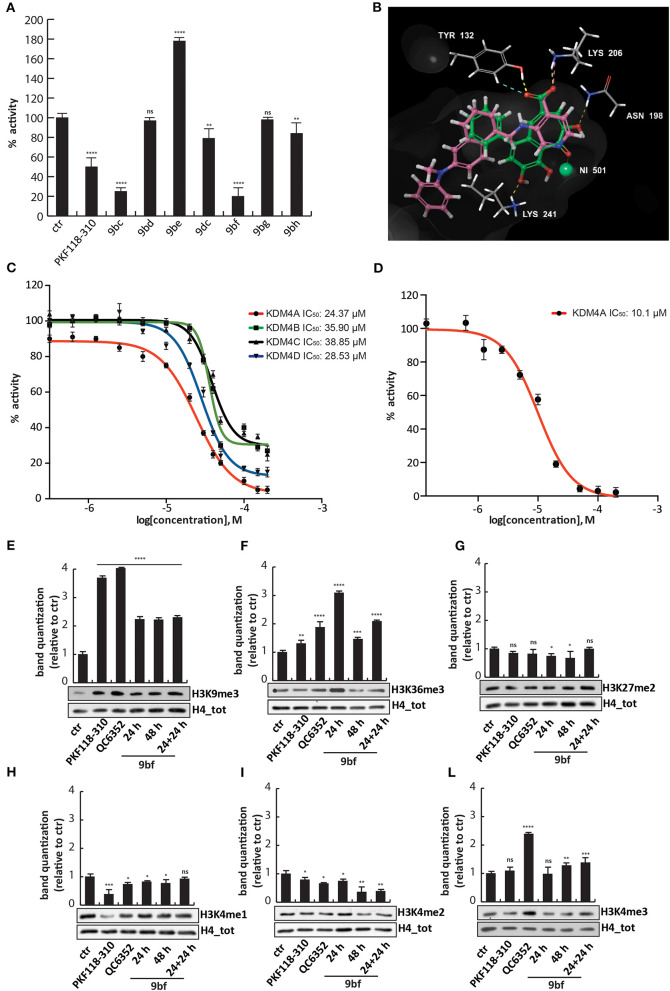
Biological characterization of selected purpurogallin analogs. **(A)** *In vitro* KDM4A enzymatic assay for the compounds at 50 μM; **(B)** Binding overlapping of **9bf** (green) with QC6352 (**7**) (pink), the co-crystallized ligand in the PDB used for our studies (5VGI). The carboxyl group of the pyridinecarboxylate of QC6352 (**7**) is perfectly overlapped with that of the benzotropolone ring, and both cores retrieved the same crucial interactions; **(C)** Estimation of **9bf** enzymatic activity against KDM4A/B/C/D using a dose-curve analysis, with concentrations ranging from 200 to 0.137 μM determined using GraphPad Prism 7 software. **(D)** AlphaLisa KDM4A assay of **9bf** at 10 different concentrations, and IC_50_ evaluation. **(E–L)** Western blot analysis for H3K9me3 **(E)**, H3K36me3 **(F)**, H3K27me2 **(G)**, H3K4me1 **(H)**, H3K4me2 **(I)**, and H3K4me3 **(L)** modifications in assays of MCF7 cells incubated for 24, 48, and 24 + 24 h (double administration) with **9bf** at 10 μM. PKF118-310 (**5**) and QC6352 (**7**) were used as positive controls for 24 h at 1 and 10 μM, respectively. H4 was used for normalization. Target levels were quantized using ImageJ software. ****p-value ≤ 0.0001, ***p-value ≤ 0.001, **p-value ≤ 0.01, *p-value ≤ 0.05, ^*ns*^*p*-value > 0.05 vs control cells.

All compounds were tested in the enzymatic assay at 50 μM to evaluate their activity. Interestingly, only **9bf** inhibited KDM4A by 80%, comparable to **9bc** (Figure 6A and Supplementary Figure 1), with an IC_50_ of 24.37 μM, similar to KDM4D (IC_50_ of 28.53 μM) but slightly higher than KDM4B/C (IC_50_ of 35.9 and 38.85 μM) (Figure 6C), suggesting a faint selectivity despite the structural homology between the enzymes.

We therefore investigated the activity against KDM4A using an orthogonal enzymatic reaction, the JMJD2A Homogeneous Assay Kit AlphaLISA® technology, based on a highly specific acceptor beads-primary antibody that recognizes demethylated biotinylated substrate. Compound **9bf** blocked the KDM4A enzyme with an IC_50_ of 10.1 μM (Figure 6D), in agreement with the greater sensibility of AlphaLISA® technology.

Docking studies were performed in order to elucidate the putative binding mode of these compounds, using Glide (release 2018-4) in extra precision mode (XP) with and without constraints (Friesner et al., 2004, 2006; Halgren et al., 2004). The output did not return docking poses for all the compounds but only for six compounds, in which **9bf** and **9bc** were prioritized with a docking score of −9.820 and −8.302 Kcal/mol respectively. As observed for other well-known KDM4 inhibitors [i.e., QC6352 (**7)** from Celgene and others] (Chen et al., 2017), compound **9bf** mimics the key 2-OG interactions (Figure 6B). In details, the carboxylic group in the heptane ring forms two H-bonds with Tyr132 and Lys206, residues normally involved in the interactions with the natural cofactor 2-OG, while the carbonyl group is involved in the Ni metal chelation, surrogate in the PDB of the natural Fe in the binding site. Furthermore, two H-bond acceptors involving hydroxyl groups were found to stabilize the binding pose of **9bf** and **9bc** (Figure 6B and Supplementary Figures 2A,B). Additionally, ligand **9bf** was prioritized with ΔG = −27.47 Kcal/mol in MM-GBSA analysis, in order to evaluate the binding free energy of the interaction.

To strengthen the putative binding mode hypothesis, an induced-fit docking, known as flexible docking, was carried out focusing on **9bf**, **9bc** and on the parent purpurogallin structure **9aa**. The classic docking studies consider the protein as rigid and during the simulations only the ligand is free to move but, as already demonstrated, the protein side chains are induced to adapt their position due to the dynamic interaction with the ligand. Induced-fit docking, adapting atoms van der Waals radius and side chain positions, takes into consideration the protein side chains as more flexible and enables the receptor to adapt its binding site based on the shape and the binding mode of the ligand (Sherman et al., 2006). XP binding mode of **9bf** and **9bc** were confirmed, with the same key interactions of Tyr 132, Lys 206 and of the metal binding with Ni, crucial for the protein activity (Supplementary Figures 2C,D). Moreover, the binding mode of the reference compound purpurogallin, **9aa**, was explored and we noticed a different pose compared with **9bc** and **9bf**. The absence of the carboxylic acid substituent seems to induce a conformational change in the ligand pose. The interaction with Tyr 132 and the metal binding were retained whereas the interaction with Lys 206 was lost (Supplementary Figure 2E). This modification, in accordance with the assays results, could suggest that the carboxylic group is crucial for the correct ligand orientation and stabilization of the interactions that could be translated into the modulation of the activity.

### Compound 9bf Inhibits KDM4A in Cells and Acts on Several Cancer Cell Lines

To investigate **9bf** activity, MCF7 cells were treated with the compound at 10 μM for 24 h, 48 h, or 24 h + 24 h in order to evaluate its effect in cells. **9bf** induced an increase in the levels of H3K36me3 and H3K9me3, two known KDM4A targets, compared to the control after 24 h and double stepwise administration (Figures 6E,F), more effectively than **9bc**.

We again assessed whether compound **9bf** was able to modulate the levels of off-targets such as H3K27me2, H3K4me1, H3K4me2, and H3K4me3. No changes in other KDM targets were observed after treatment with **9bc**, suggesting the selectivity of this compound for KDM4A (Figures 6G–L) (Berry and Janknecht, 2013).

**Figure 7:**
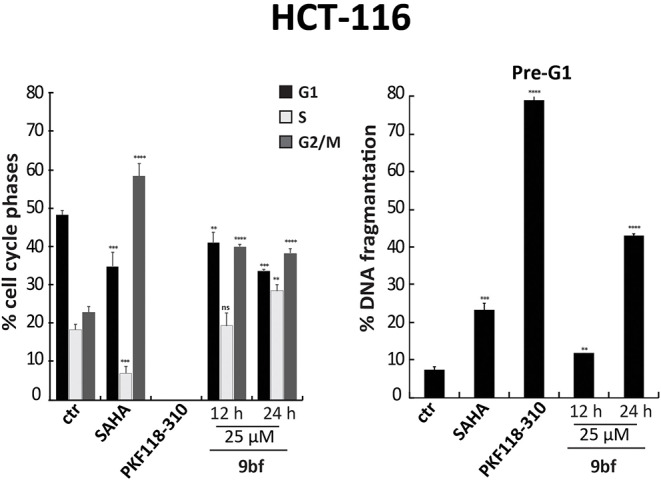
Cell cycle analysis of **9bf**. HCT-116 cells were treated with **9bf** for 12 and 24 h at 25 μM. SAHA and PKF118-310 (**5**) were used as positive controls for 24 h at 5 and 10 μM, respectively. ****p-value ≤ 0.0001, ***p-value ≤ 0.001, **p-value ≤ 0.01, *p-value ≤ 0.05, ^*ns*^*p*-value > 0.05 vs control cells.

Next, we performed cell cycle analyses on HCT-116 cells in order to evaluate the ability of **9bf** to induce cell cycle variation and cancer cell death (Figure 7). In these experiments, SAHA (Zhou et al., 2001) and PKF118-310 (**5**) (Franci et al., 2017) were used as controls. Our data show a significant induction of HCT-116 cell death by **9bf**, revealed by pre-G1 phase analysis. Specifically, in HCT-116 cells **9bf** was able to induce a blockage in G2/M phase, accompanied by a significant increase (up to ~40%) in cell population at pre-G1 phase. This result is comparable to the activity of SAHA, a well-known anticancer drug, which exhibited its antitumoral activity through G2/M phase accumulation after 24 h at a concentration of 5 μM, especially in hematological cancer cells.

**Figure 8:**
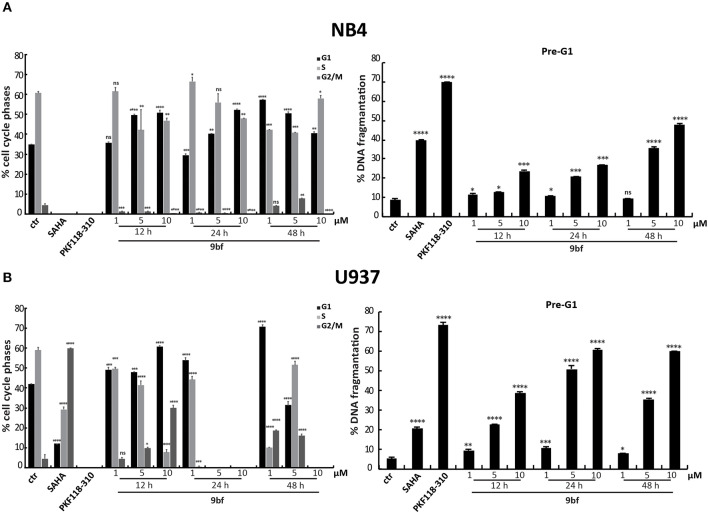
Cell cycle analysis of **9bf** on hematological cell lines. NB4 **(A)** and U937 **(B)** cells were treated with **9bf** for 12, 24, and 48 h at 1, 5, and 10 μM. SAHA and PKF118-310 (**5**) were used as positive controls for 24 h at 5 and 10 μM, respectively. ****p-value ≤ 0.0001, ***p-value ≤ 0.001, **p-value ≤ 0.01, *p-value ≤ 0.05, ^*ns*^*p*-value > 0.05 vs control cells.

We also evaluated the effect of **9bf** in two leukemia cell lines. Acute promyelocytic leukemia NB4 and histiocytic lymphoma U937 cells were treated in three doses and at three different times in order to better assess its activity. Given the sensitivity to treatment of these cell lines compared to colon and breast cancer cells, lower doses (1–10 μM) were used. Our results show that **9bf** acted in a time- and dose-dependent manner, strongly inducing cell death especially in U937 cells already at 5 μM after 24 h of treatment (Figures 8A,B).

**Figure 9:**
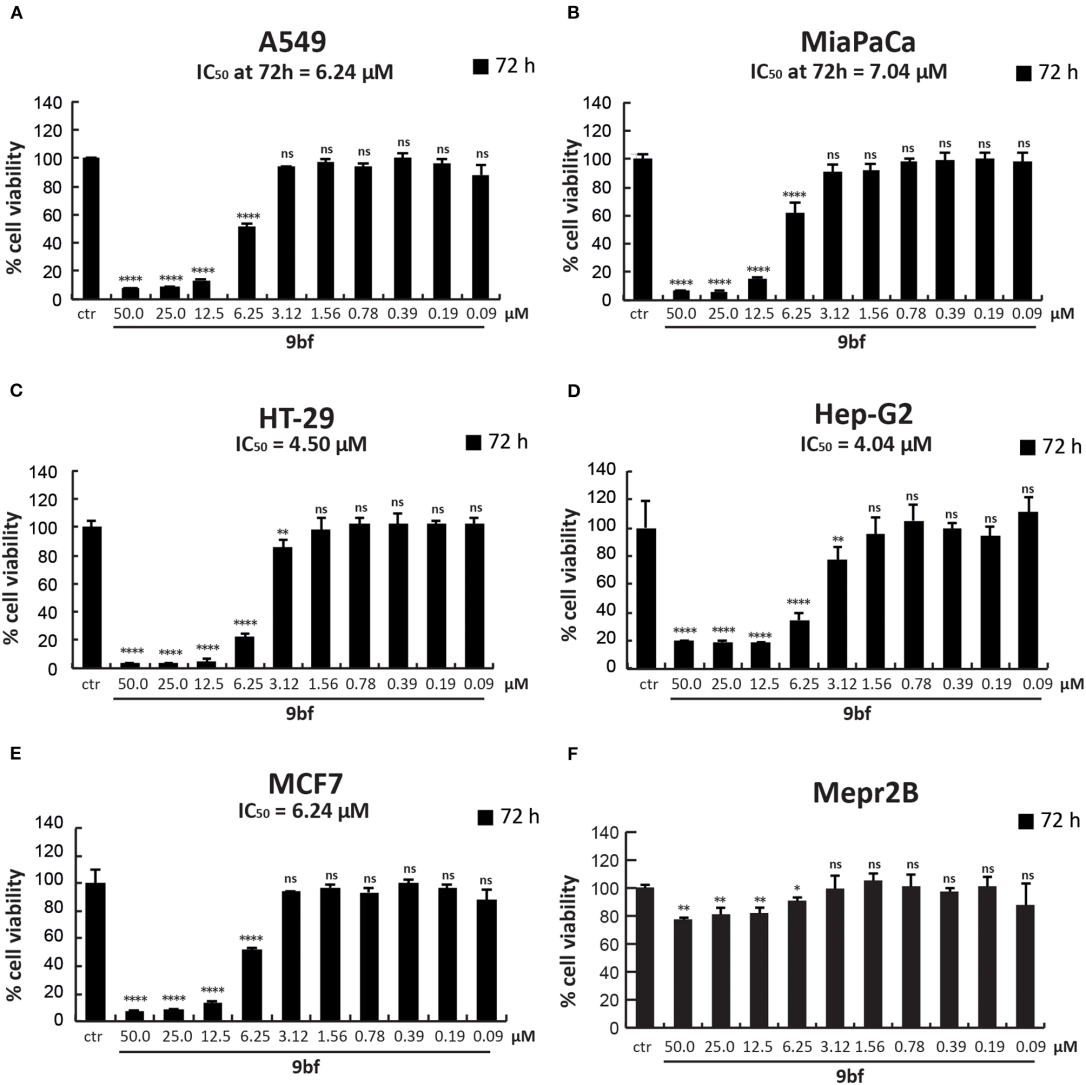
Cell viability study of **9bf**. A549 **(A)**, MiaPaCa **(B)**, HT-29 **(C)**, Hep-G2 **(D)**, MCF7 **(E)**, and Mepr2B **(F)** cell lines treated with **9bf** (50–0.09 μM) for 72 h. Absorbance was determined at a wavelength of 570 nm with a TECAN M-200 reader. ****p-value ≤ 0.0001, ***p-value ≤ 0.001, **p-value ≤ 0.01, *p-value ≤ 0.05, ns *p*-value > 0.05 vs control cells.

To further investigate its anticancer activity, **9bf** was tested in several solid cancer cell lines (Figure 9) and its activity was evaluated by MTT colorimetric assay. A549 (lung; A), MiaPaCa (pancreas; B), HT-29 (colon; C), Hep-G2 (liver; D), and MCF7 (breast; F) cells were treated with **9bf** at 10 different concentrations (from 50 to 0.09 μM) for 72 h. Our data show that compound **9bf** was able to induce a strong cell death in all cancer cell lines, as shown by the IC_50_ values (calculated using GraphPad Prism 7 software) ranging from 7.04 μM to 4.04 μM. This tight range of IC_50_ values was in line with the RNA expression analysis of KDM4A in these cancer cells (Supplementary Figure 3; http://ist.medisapiens.com/#ENSG00000066135).

Interestingly, compound **9bf** did not show any antiproliferative effects in immortalized Mepr2B cells, a normal mesenchymal cell line (Figure 9F) (Miceli et al., 2013). Only at the highest doses (50 and 25 μM), did **9bf** induce a reduction in cell proliferation of ~20%. These findings suggest that **9bf** exerts a cancer-selective antiproliferative effect with high selectivity.

## Discussion

KDM4A is known to be involved in several cellular functions, including embryonic development, cell cycle regulation, and DNA damage response (Guerra-Calderas et al., 2015). Recently, KDM4A was shown to be implicated in pathological states such as cancer (Berry and Janknecht, 2013). Specifically, overexpression of this enzyme was found in colon, breast, and prostate cancer cells. Therefore, efforts to develop new inhibitors able to block KDM4A activity and inhibit cancer cell proliferation are increasing. Purpurogallin **9aa**, already described as an anti-inflammatory, antioxidant, and anticancer agent (Wu et al., 1996; Kitada et al., 2003; Leone et al., 2003; Sang et al., 2004; Cheng et al., 2012; Xie et al., 2019), was found to be a KDM4A inhibitor. Based on the structure of **9aa**, we synthesized a set of derivatives and analogs by changes on the starting phenol ring. Among these molecules, compounds **9ac** and **9bc** showed a better enzymatic activity. In particular, **9bc** inhibited KDM4A more strongly than PKF118-310 (**5**), a known KDM4A inhibitor. In MCF7, **9ac** better than **9bc** selectively increased the methylation levels of lysine 9 and 36, targets of this enzyme, and did not change the levels of all off-targets tested. However, none of the compounds induced cell death.

These precedents led to the development of a new compound, namely **9bf**. This analog shares the common benzotropolone scaffold and incorporates an additional aromatic ring at position R_1_ of the catechol unit that increases lipophilicity, and a carboxylic acid at position R_2_ of the pyrogallol fragment. This molecule strongly inhibited KDM4A in *in vitro* assays with an IC_50_ of 24.37 and 10.1 μM, and in breast cancer cells increased the levels of H3K9me3 and H3K36me3, specific targets of KDM4A, already after 24 h of treatment at 10 μM. Given the structural homology among the enzymes belonging to the KDM4 subfamily, **9bf** also inhibits KDM4B/C/D but with a greater IC_50_, demonstrating the selectivity of this compound toward KDM4A (Figure 6C). It also exerted robust anticancer activity in HCT-116 colon and in both U937 and NB4 hematological cancer cells, inducing cell death in a time- and dose-dependent manner.

Excitingly, **9bf** displayed high toxicity in several solid tumor cell lines, with IC_50_ values from 7.04 to 4.04 μM, but low activity in normal cells, potentially making it an excellent chemotherapeutic agent. Our findings suggest that further development of this compound and its derivatives may lead to the identification of new therapeutic antitumor agents that act by strongly inhibiting KDM4A.

To summarize, in this work we describe the synthesis and biological characterization of a new family of epigenetic modulators based on the structure of the natural product purpurogallin. Among the analogs synthesized, we identified compound **9bf** as a potent modulator of the epigenetic enzyme KDM4A, with promising antiproliferative effects in different cell systems. Our findings highlight the key structural role of the benzotropolone core of these molecules in inhibiting the enzyme, as well as the increased inhibitory activity exhibited by the carboxylic acid at C8 containing an aryl group at C1, which appear to induce a better binding of these benzotropolone derivatives to the ligand-binding pocket of Jumonji C domain-containing KDMs.

## Data Availability Statement

All datasets generated for this study are included in the article/Supplementary Material.

## Author Contributions

ÁL and RÁ planned and coordinated, and JS planned and performed the organic chemical synthesis of the compounds. FS and CP performed biological experiments, and data analysis and interpretation. UP, JL, and AP performed and contributed to the conception of binding *in silico* studies. AN and LA contributed to the conception of the biological experiments. All authors contributed to manuscript revision, read and approved the submitted version.

## Conflict of Interest

CP was employed by the company EPI-C srl. The remaining authors declare that the research was conducted in the absence of any commercial or financial relationships that could be construed as a potential conflict of interest.
