# Supplementary material for: A New Family of Jumonji C Domain-Containing KDM Inhibitors Inspired by Natural Product Purpurogallin
Source: Front Chem. 2020 May 25;8:312. doi: 10.3389/fchem.2020.00312 (PMC7261929; doi:10.3389/fchem.2020.00312)
Supplement: Supplementary file 1 [file Data_Sheet_1.docx]

# Supplementary Material

A new family of Jumonji C domain-containing KDM inhibitors inspired by natural product purpurogallin

José A. Souto^1,a^, Federica Sarno^2,a^, Angela Nebbioso^2^, Chiara Papulino^3^, Rosana Álvarez^1^, Jessica Lombino^4^, Ugo Perricone^4^, Alessandro Padova^4^, Lucia Altucci^2,*^, and Ángel R. de Lera^1*^

^1^ Departamento de Química Orgánica, Facultade de Química and Centro de Investigacións Biomédicas (CINBIO). Universidade de Vigo, 36310 Vigo, Spain. [qolera@uvigo.es](mailto:qolera@uvigo.es).

^2^ Dipartimento di Medicina di Precisione, Università degli Studi della Campania “L. Vanvitelli”, Napoli, Italy.

^3^ Epi-C srl, Napoli, Italy.

^4^ Fondazione Ri.MED, Palermo, Italy

ª Those authors contributed equally to the work.

*** Correspondence:**Lucia Altucci
[lucia.altucci@unicampania.it](mailto:lucia.altucci@unina2.it)

Ángel R. de Lera
[qolera@uvigo.es](mailto:qolera@uvigo.es)


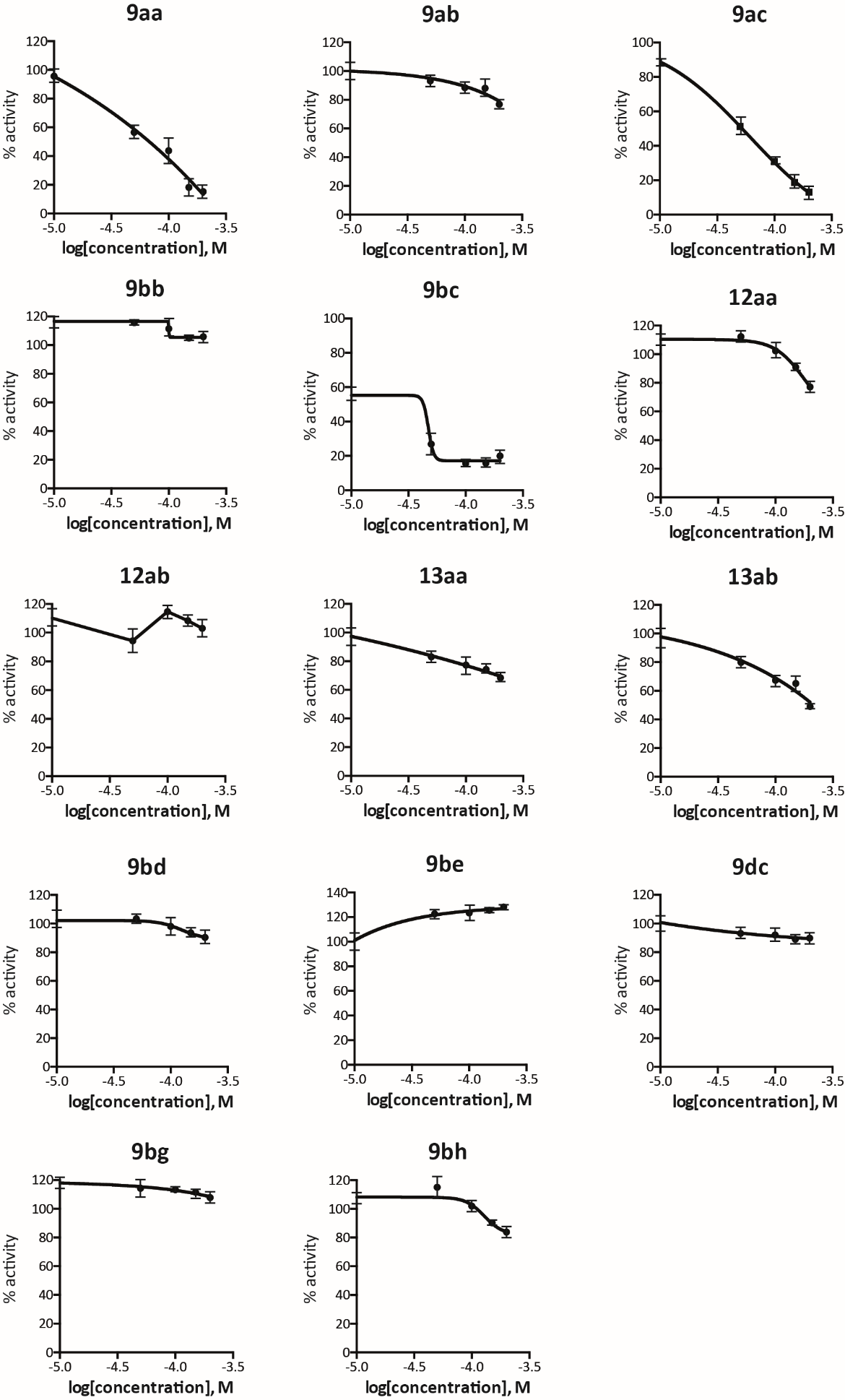


**Supp. Figure 1**: *In vitro* KDM4A enzymatic assay of all compounds at 200, 150, 100, 50, and 10 µM.

**Supp. Figure 2:** table of binding sites of **9bf**, **9bc** and **9aa** compounds in KDM4A.


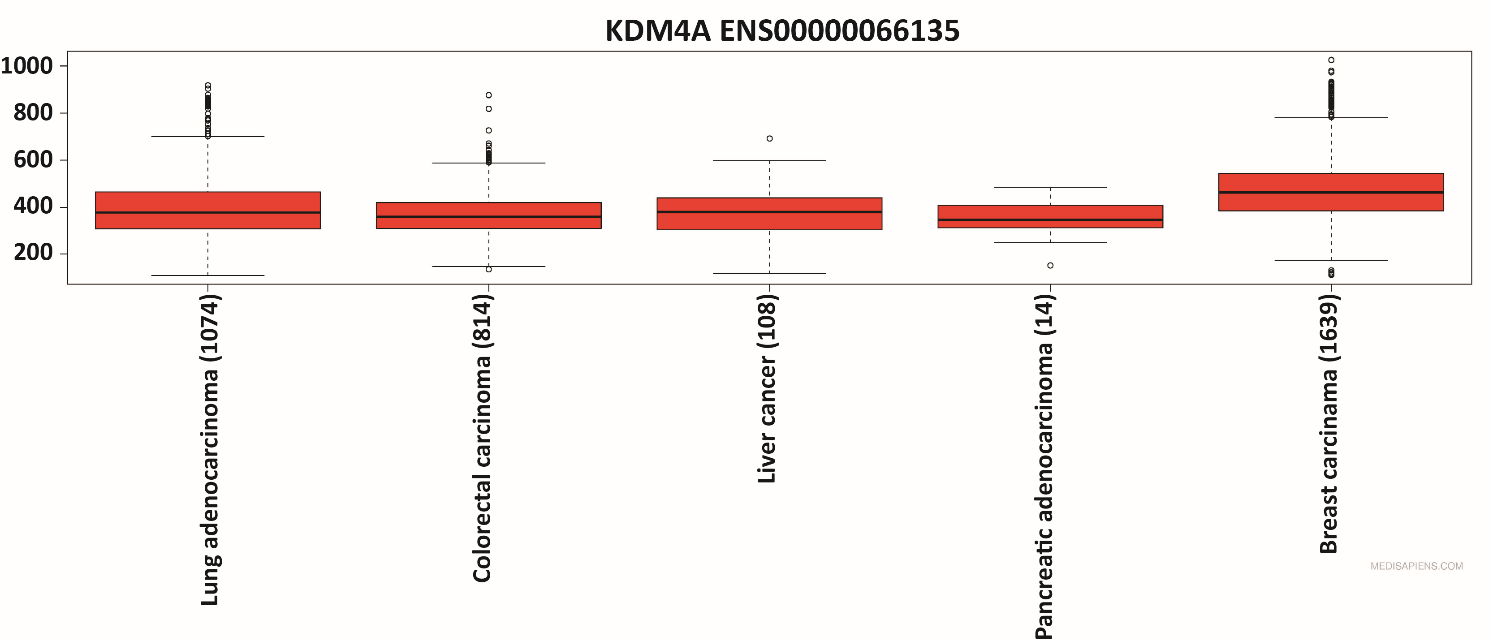


**Supp. Figure 3**: *KDM4A* expression. Analysis of *KDM4A* gene expression levels in lung, colorectal, liver, pancreatic, and breast cancer. Values were obtained by ITS online *in silico* transcriptomics.

**Copies of NMR spectroscopic data**

**9aa**

**9ab**

**9ac**

**9bb**

**9bc**

**12aa**

**12ab**

**13aa**

**13ab**

**15c**


**9cc**

**9cd**

**9ce**

**18cc**

**19cf**

**19cg**

**19ch**


**20dc**

**9bd**

**9be**

**9bf**

**9bg**

**9bh**

**9dc**
